# Inactivated vaccine with glycyrrhizic acid adjuvant elicits potent innate and adaptive immune responses against foot-and-mouth disease

**DOI:** 10.3389/fmicb.2023.1289065

**Published:** 2023-10-31

**Authors:** Seokwon Shin, Hyeong Won Kim, Mi-Kyeong Ko, So Hui Park, Su-Mi Kim, Jong-Hyeon Park, Min Ja Lee

**Affiliations:** Animal and Plant Quarantine Agency, Gimcheon-si, Gyeongsangbuk-do, Republic of Korea

**Keywords:** glycyrrhizic acid, adjuvant, foot-and-mouth disease, vaccine, innate and adaptive, immune response

## Abstract

**Background:**

Foot-and-mouth disease (FMD) is an extremely contagious viral disease that is fatal to young animals and is a major threat to the agricultural economy by reducing production and limiting the movement of livestock. The currently commercially-available FMD vaccine is prepared using an inactivated viral antigen in an oil emulsion, with aluminum hydroxide [Al(OH)_3_] as an adjuvant. However, oil emulsion-based options possess limitations including slow increases in antibody titers (up to levels adequate for defense against viral infection) and risks of local reactions at the vaccination site. Further, Al(OH)_3_ only induces a T helper 2 (Th2) cell response. Therefore, novel adjuvants that can address these limitations are urgently needed. Glycyrrhizic acid (extracted from licorice roots) is a triterpenoid saponin and has great advantages in terms of price and availability.

**Methods:**

To address the limitations of the currently used commercial FMD vaccine, we added glycyrrhizic acid as an adjuvant (immunostimulant) to the FMD bivalent (O PA2 + A YC) vaccine. We then evaluated its efficacy in promoting both innate and adaptive (cellular and humoral) immune reactions *in vitro* [using murine peritoneal exudate cells (PECs) and porcine peripheral blood mononuclear cells (PBMCs)] and *in vivo* (using mice and pigs).

**Results:**

Glycyrrhizic acid has been revealed to induce an innate immune response and enhance early, mid-, and long-term immunity. The studied bivalent vaccine with glycyrrhizic acid increased the expression of immunoregulatory genes such as pattern-recognition receptors (PRRs), cytokines, transcription factors, and co-stimulatory molecules.

**Conclusion:**

Collectively, glycyrrhizic acid could have utility as a novel vaccine adjuvant that can address the limitations of commercialized FMD vaccines by inducing potent innate and adaptive immune responses.

## 1. Introduction

Foot-and-mouth disease (FMD) is an acute viral disease that spreads rapidly and is highly contagious (Grubman and Baxt, 2004). It is prevalent in over 100 countries globally (Jamal and Belsham, 2013). It affects cloven-hooved livestock such as swine, sheep, cattle, buffalo, and goats, with significant negative impacts on the livestock industry (Grubman and Baxt, 2004). The FMD virus (FMDV) first enters the oropharynx of swine. Thereafter, it forms a vesicle from the snout toward the breast, nipples, or feet for infection. Animals that have contracted the virus display symptoms such as increased salivation, elevated body temperature, and decreased milk production. Infected animals are at risk of developing secondary infections, experiencing weight loss, and exhibiting reduced productivity over time. This can lead to reduced livestock productivity and high neonatal mortality (Arzt et al., 2011; Stenfeldt et al., 2014; Fukai et al., 2015b; Casey-Bryars et al., 2018). Due to the rapid spread of FMD, regions or countries with endemic FMD should aim to quickly contain any viral spread, the failure of which can lead to national restrictions on animal products and enormous economic losses (Rodriguez and Grubman, 2009; Casey-Bryars et al., 2018).

FMDV is a single-stranded positive-sense RNA virus categorized under the genus *Aphthovirus* within the family *Picornaviridae* (Jamal and Belsham, 2013). Its capsid is made up of 60 copies of structural proteins (SP), namely, VP1, VP2, VP3, and VP4 (Grubman and Baxt, 2004). The VP1 protein is known to be a key target in the prevention of FMD since it has the strongest immunogenicity, many neutralizing sites of the virus, a serotype-determining area, and is closely related to host cell-virus binding (Cheung et al., 1983; Parry et al., 1990; Jackson et al., 2003). FMDV has seven serotypes, namely, O, A, Asia1, South African Territories (SAT)1, SAT2, SAT3, and C. These serotypes do not induce cross-protection toward one other, and each serotype has multiple topotypes (Mason et al., 2003; Grubman and Baxt, 2004). Consequently, to protect against each serotype, appropriate vaccines must be developed and employed accordingly (Lee et al., 2020). Countries with endemic FMD, where many animals are still affected by the disease, need to introduce vaccines to prevent its spread. Vaccines alone are not a fundamental solution and do not completely address the losses related to FMD; nevertheless, vaccination, before FMD occurs, is likely to improve immunity in livestock animals (Rodriguez and Grubman, 2009; Casey-Bryars et al., 2018).

In the early 1930s, formalin was discovered to be capable of killing live FMDV. During the 1960s, the introduction of an FMD vaccine [which used formalin-inactivated FMDV cultured in baby hamster kidney (BHK) cells] resulted in a significant decrease in the prevalence of the disease across various European nations (Leforban and Gerbier, 2002). Currently, Korea supplies vaccines containing antigen-enhanced O, A, and Asia1 to prevent viruses that can simultaneously infect livestock farms vulnerable to FMD, and these vaccines induce high antibody titers. However, FMD remains a problem in numerous countries throughout the Middle East, South America, Africa, and Asia (Barteling, 2002). In addition, currently commercialized FMD vaccines have the following disadvantages; (1) in the case of inactivated virus vaccines, it is difficult to provide substantial disease protection until antibodies are formed post-vaccination; (2) pigs vaccinated with inactivated vaccines develop relatively lower antibody titers than cattle, and there are large inter-individual differences; and (3) side effects such as granulomas and fibrosis at the site of injection can be caused by oil emulsion-based formulations and are emerging as serious problems in pigs (Di Pasquale et al., 2015; Kamel et al., 2019). Numerous attempts to develop improved vaccines have been made. Since FMDV shows the highest antigenicity in its complete form, inactivated vaccines are currently the most efficacious. However, to overcome the existing limitations of inactivated viral vaccines, research on adjuvants capable of improving the immunogenicity of vaccines is currently being conducted.

Adjuvants are agents incorporated into vaccines to regulate and enhance the immune response. Many adjuvants have been isolated from natural products. The first research on adjuvants began in 1925, when tapioca starch was mixed with diphtheria toxoid and effectively increased antigen-specific antibody production (Ramon, 1925, 1926). Aluminum is currently used as a representative adjuvant; in 1926, aluminum-deposited diphtheria toxoid was shown to possess better immunogenicity than the toxoid alone, and aluminum has been in use for over 80 years to date (Hogenesch, 2013). Aluminum hydroxide [Al(OH)_3_], the most commonly used chemical adjuvant, can induce antibody-mediated T helper 2 (Th2) responses which strongly stimulate IgE production, exerting a potent inflammatory effect at the injection site (Hem and Hogenesch, 2007; Jiang et al., 2018). However, since the cellular immune response mediated by CD8^+^ and CD4^+^ T lymphocytes is increasingly recognized to be equally critical for acquired defense in vaccinated animals, many research efforts are now attempting to identify adjuvants that elicit Th1 and Th2 immune responses, as well as innate and adaptive immune responses simultaneously (Guzman et al., 2010; Carr et al., 2013).

Glycyrrhizic acid, a representative component of licorice, is a triterpenoid-based material and comprises approximately 5% of licorice roots. Glycyrrhizic acid is generally recognized as safe and has been approved by the Food and Drug Administration (FDA) as a food additive in the United States since 1985 (Bailly and Vergoten, 2020). In 1925, saponin was found to significantly increase antibody responses to tetanus. Further, saponin can be used as an adjuvant since it has been found to induce a cellular immune response by increasing helper and cytotoxic T-cell responses as well as the antibody response (Ramon, 1925; Egerton et al., 1978). Saponin has been approved as a food and is widely used in medicines and cosmetics due to its various pharmacological effects, including its antioxidant, antiviral, anti-ulcer, and antibacterial activities. Glycyrrhizic acid is a major plant-derived metabolite that is still used in the long-term treatment of human immunodeficiency virus (HIV) infection owing to its low potential for drug resistance and few side effects (De Clercq, 1995; Vlietinck et al., 1998). Recent studies have shown that glycyrrhizic acid is a promising anti-severe acute respiratory syndrome (SARS)-CoV-2 drug candidate, either alone or in conjunction with other therapeutics (Bailly and Vergoten, 2020).

Components isolated from plant-derived natural products have been used as potential chemotherapeutic, chemopreventive, and anti-inflammatory agents over the last 30 years (Surh, 2002). Considering that the ideal adjuvant should be easy to obtain, safe, and easy to store, glycyrrhizic acid holds promise for this application. Although there are many studies on the effects of glycyrrhizic acid, clear results on its use as an adjuvant in the FMD vaccine are yet to be reported. The objective of this research was to explore the impact of glycyrrhizic acid as an adjuvant, overcome the drawbacks of current vaccines, and elicit an enhanced host immune response. Furthermore, we attempted to elucidate the mechanism of glycyrrhizic acid-mediated immune response induction in hosts.

## 2. Materials and methods

### 2.1. Glycyrrhizic acid

Glycyrrhizic acid ammonium salt [from glycyrrhiza roots (licorice)] was purchased from Sigma-Aldrich (Sigma-Aldrich, St. Louis, MO, USA).

### 2.2. Cells, virus, purification of inactivated viral antigens, and preparation of test bivalent vaccine

ZZ-R (fetal goat tongue) cells were grown in DMEM/F12 (Lonza, Basel, Switzerland), and BHK-21 and LF-BK (porcine kidney) cells were grown in DMEM (Lonza, Walkersville, MD, USA) supplemented with 1% antibiotic-antimycotic and 10% fetal bovine serum (FBS) (Gibco, Waltham, MA, USA) at 37°C in 5% CO_2_. FMDV O PA2 and A YC antigens were purified according to the method described by Lee et al. (2020). Briefly, when full cytopathic effects (CPE) were noted, viruses (O PA2, A YC; grown in BHK-21 suspension cells) were collected via freezing and thawing. Once the cellular debris was eliminated via centrifugation at 12,000 rotations per minute (rpm) for 20 minutes (min), the viruses were exposed to 0.003 mM of binary ethyleneimine (Sigma-Aldrich) at 26°C for 24 hour (h). The inactivated virus within the transparent clear culture was allowed to precipitate overnight in 7.5% polyethylene glycol (PEG) 6000 and 2.3% NaCl at 4°C (Bahnemann, 1975). The obtained pellets were suspended in Tris-KCl (TK) buffer and further subjected to purification via centrifugation (using a sucrose gradient of 15–45% in TK buffer) at 30,000 rpm for 4 h at 4°C on an SW41Ti rotor (Beckman Coulter, Brea, CA, USA); viral antigen concentrations were assessed by spectrophotometric (Biophotometer, Eppendorf, Hamburg, Germany) analysis at a wavelength of 259 nm. The purified antigens (146S particle) were fixed onto carbon-coated copper grids and observed through a transmission electron microscope (TEM) (Hitachi H7100FA, Tokyo, Japan). The bivalent vaccine formulation for the positive control (PC) group in the mouse experiment included purified antigens isolated from O PA2 (15 μg/dose/mL; 1/40 of the dose for pigs) and A YC (15 μg/dose/mL; 1/40 of the dose for pigs), 10% Al(OH)_3_, ISA 206 (50% w/w; Seppic, Paris, France), and 15 μg/dose/mouse Quil-A (InvivoGen, San Diego, CA, USA). 100 μg glycyrrhizic acid ammonium salt (Sigma-Aldrich) was also included in the test vaccine for the experimental (Exp.) group. The test bivalent vaccine formulation used in the pig experiment included purified antigens isolated from O PA2 (15 μg/dose/mL) and A YC (15 μg/dose/mL), 10% Al(OH)_3_, ISA 206 (50% w/w; Seppic), and 150 μg/dose/pig Quil-A (InvivoGen). One mg glycyrrhizic acid ammonium salt (Sigma-Aldrich) was also included in the test vaccine for the Exp. group. All experiments related to FMDV were conducted in the Animal and Plant Quarantine Agency (APQA) under biosafety level 3 (BSL-3) conditions.

### 2.3. Mice and pigs

Sex- and age-matched wild-type C57BL/6 mice (females, 6–7 weeks old) were obtained from KOSA BIO Inc. (Seongnam-si, Gyeonggi-do, Korea), and FMD-antibody-seronegative pigs (Landrace, 8–9 weeks old) were supplied by BARON BIO Inc. (Uiseong-gun, Gyeongsangbuk-do, Korea). All mice and pigs were allowed at least 1 week to acclimatize to the laboratory environment before being used in experiments. All mice and pigs were accommodated in microisolator enclosures and were allowed unrestricted access to water and food in a dedicated pathogen-free BSL-3 animal (ABSL3) facility at the APQA. The housing facility maintained a relative humidity of approximately 50%, a temperature of 22°C, and a 12 h:12 h light:dark cycle. Investigations were conducted following institutional protocols and regulations and authorized by the Ethics Committee of the APQA (accreditation number: IACUC-2022-670 and IACUC-2023-753).

### 2.4. Isolation and culture of murine peritoneal exudate cells (PECs) and porcine peripheral blood mononuclear cells (PBMCs)

Naïve mice (6–7 weeks old, *n* = 40) were euthanized via CO_2_ inhalation. The peritoneal cavities of the mice were then rinsed with 5 mL Ca^2+^/Mg^2+^/free DPBS (Gibco), and the resulting peritoneal lavage fluid was pelleted by centrifugation at 400 × *g* for 10 min at 4°C. The pelleted murine PECs were resuspended and counted using a Bio-Rad TC20 Automated Cell Counter (Bio-Rad, Hercules, CA, USA). All obtained cells were isolated in the fresh state immediately prior to use. None of the experiments involved the use of cryopreserved cells. Subsequently, purified PECs were cultured in a complete medium composed of Roswell Park Memorial Institute (RPMI) 1640 (Gibco) supplemented with 0.05 mM 2-beta-mercaptoethanol (Sigma-Aldrich), 3 mM L-glutamine (Sigma-Aldrich), 10% fetal calf serum (HyClone, Logan, UT, USA), 100 U/mL penicillin/streptomycin (Sigma-Aldrich), and 10 mM HEPES (Sigma-Aldrich) and incubated at 37°C under 5% CO_2_. Porcine PBMCs were acquired from pigs (8–9 weeks old, *n* = 3 for the evaluation of IFNγ secretion by ELISpot, *n* = 5–6/group for the validation of gene expression by qRT-PCR) that tested negative for FMD antibodies. They were then purified from whole blood according to the procedure described by Lee et al. (2020) and Jo et al. (2021). Whole blood (20 mL/donor) was collected in BD Vacutainer heparin tubes (BD, Becton, Dickinson and Company, Franklin Lakes, NJ, USA). Porcine PBMCs were isolated through gradient centrifugation using Histopaque solution (Sigma-Aldrich). The remaining red blood cells (RBC) were eliminated with ammonium–chloride–potassium (ACK) lysing buffer (Gibco). Porcine PBMCs were suspended in Ca^2+^/Mg^2+^-free DPBS (Gibco). Cell quantification was performed with a Bio-Rad TC20 Automated Cell Counter (Bio-Rad). The separated porcine PBMCs were then suspended in RPMI-1640 (Gibco) medium supplemented with 100 U/mL penicillin/streptomycin (Sigma-Aldrich), 10 mM HEPES (Sigma-Aldrich), 3 mM L-glutamine (Sigma-Aldrich), and 10% FBS (Gibco). All purified cells were cultured in a moist environment at 37°C with 5% CO_2._

### 2.5. Glycyrrhizic acid-mediated cell viability assay and IFNγ ELISpot assay in PECs and porcine PBMCs *in vitro*

BHK-21, LF-BK, and ZZ-R cell lines, murine PECs, and porcine PBMCs were used in the cell viability assay. BHK-21, LF-BK, and ZZ-R cells (2 × 10^4^ cells/well) were cultured in a 96-well microplate and incubated for 48 h in 5% CO_2_ at 37°C. Isolated murine PECs and porcine PBMCs (1 × 10^5^ cells/well) were seeded in a 96-well microplate and stabilized for 1 h in 5% CO_2_ at 37°C. After incubation, the media was changed and BHK-21, LF-BK, and ZZ-R cells, murine PECs, and porcine PBMCs were treated with glycyrrhizic acid (0, 0.625, 1.25, 2.5, or 5 μg/mL) for 4 h. Cell viability was determined using an MTS (inner salt)-based colorimetric assay. Investigations were performed by introducing a small quantity of CellTiter® 96 AQueous One Solution Reagent (Promega, Madison, WI, USA) directly to the culture wells, incubating the plates for 4 h in 5% CO_2_ at 37°C, and subsequently measuring the absorbance at 490 nm with a Hidex 300SL spectrophotometer (Hidex, Turku, Finland). Commercial ELISpot assay kits (R&D Systems, Minneapolis, MN, USA) were used to analyze glycyrrhizic acid-induced IFNγ secretion, with or without inactivated O PA2 and A YC antigens, according to the manufacturer’s instructions. In brief, isolated murine PECs (5 × 10^5^ cells/well) or porcine PBMCs (5 × 10^5^ cells/well) were incubated in a 96-well PVDF microplate which was backed with a monoclonal capture antibody that targets either mouse or porcine IFNγ, which had been pre-coated onto the plate. Subsequently, the cells were exposed to inactivated FMDV (O PA2 and A YC) antigens at a concentration of 2 μg/mL (final concentration), either with or without 0.625, 1.25, 2.5, or 5 μg/mL glycyrrhizic acid in a humidified incubator for 18 h in 5% CO_2_ at 37°C. PBS and 2 μg/mL inactivated FMDV (O PA2 or A YC) antigen were used for the negative control (NC) and PC, respectively. The plates were rinsed with wash buffer and then incubated overnight at 4°C with biotinylated anti-mouse IFNγ (1:119) or anti-porcine IFNγ (1:119) antibodies. Thereafter, they were treated with AP-conjugated streptavidin (1:119) for 2 h at room temperature (RT, approximately 25°C). The plates were rinsed, developed with 5-bromo-4-chloro-3′ indolyl phosphate p-toluidine salt (BCIP)/nitro blue tetrazolium chloride (NBT), and quantified using an ImmunoSpot ELISpot reader (AID iSpot Reader System; Autoimmune Diagnostika GmbH, Strassberg, Germany). Data were reported as the number of spot-forming cells (SFC).

### 2.6. Effects of glycyrrhizic acid alone on inducing host defenses against FMDV infection in mice

Prior to evaluating the adjuvant efficacy of glycyrrhizic acid, we investigated the host defense induced by glycyrrhizic acid alone (without FMDV antigen) against FMDV infection in mice. The following challenge viruses were used; FMDV type O [O/VET/2013 (ME-SA topotype, GenBank Accession No. MF947143.1)] and FMDV type A [(A/Malay/97, SEA topotype, GenBank Accession No. KJ933864)]. Since there were two types of viruses used in the challenge, C57BL/6 mice (females, 6–7 weeks old, *n* = 5/group) were divided into two sets and then into two groups; NC, Exp. I on 3 days-post injection (dpi) challenged group; NC, Exp. II on 7 dpi challenged group for each virus. After intramuscular (I.M.) injection, all mice were challenged with 100 LD_50_ of O/VET/2013 or A/Malay/97 viruses via intraperitoneal (I.P.) injection on 3 or 7 dpi, and the survival rate and body weights were observed for 7 days post-challenge (dpc). The Exp. I and Exp. II groups received an injection of 100 μg glycyrrhizic acid/100 μL PBS, and the NC groups were given an equivalent volume of PBS via the same route.

### 2.7. Efficacy of FMD vaccine containing glycyrrhizic acid in inducing adjuvant-mediated host defense in mice

To evaluate the potential of glycyrrhizic acid as a vaccine adjuvant and the initial protective effect of the vaccine containing glycyrrhizic acid against viral infection, we performed challenge experiments in mice. C57BL/6 mice (females, 6–7 weeks old, *n* = 5/group) were divided into two sets for each virus and then into three groups (NC, PC, and Exp.). After I.M. vaccination, all mice were challenged with 100 LD_50_ of FMDV O/VET/2013 or A/Malay/97 virus via I.P. injection at 7 days post-vaccination (dpv), and observed for 7 dpc. The PC group was given the test bivalent vaccine, the Exp. group was given the test bivalent vaccine with glycyrrhizic acid, and the NC group was given an equivalent volume of PBS via the same route.

### 2.8. Efficacy of FMD vaccine containing glycyrrhizic acid in inducing adjuvant-mediated early, mid-term, and long-term immune responses in mice

To assess the efficacy of glycyrrhizic acid in eliciting innate and adaptive (cellular and humoral) immune responses as an FMD vaccine adjuvant, we performed mice experiments as follows. C57BL/6 mice (females, 6–7 weeks old, *n* = 5/group) were divided into three groups (NC, PC, Exp.). Following I.M. injection of the test bivalent vaccine, blood samples were collected at 0, 7 (early), 28 (mid-term), 56, and 84 (long-term) dpv to monitor antibody titers and virus neutralization (VN) titers using structural protein (SP) ELISA and VN tests, respectively. The serum samples were preserved at −80°C until further use.

### 2.9. Efficacy of FMD vaccine containing glycyrrhizic acid in inducing adjuvant-mediated early, mid-term, and long-term immune responses in pigs

FMD-antibody-seronegative pigs (8–9 weeks old, *n* = 5–6/group) were used for the experiments. Animals were randomly divided into three groups (NC, PC, Exp.). After the initial I.M. injection for primary vaccination, booster doses were given via the same route on 28 dpv. Blood samples were collected at 0, 7, 14 (early), 28, 42 (mid-term), 56, and 84 (long-term) dpv to measure the antibody titers and VN titers via SP ELISA and VN tests, respectively. The serum samples were preserved at −80°C until further use.

### 2.10. Serological assay

#### 2.10.1. Structural protein (SP) ELISA

To identify serum antibodies against structural proteins, we utilized PrioCheck^™^ FMDV type A (Prionics AG, Switzerland) kits and VDPro^®^ FMDV type O (Median Diagnostics, Chuncheon-si, Gangwon, Korea) kits. Optical density readings from the ELISA plates were transformed into a percentage inhibition (PI) value. When the PI value ≥50% for the PrioCheck^™^ FMDV kit or ≥ 40% for the VDPro^®^ FMDV kit, the animals were classified as antibody-positive.

#### 2.10.2. VN tests

VN tests were carried out following guidelines from the World Organization for Animal Health (WOAH). Serum from the vaccinated animals was inactivated via heating at 56°C for 30 min in a water bath. The cell density was adjusted to achieve a 70% monolayer, and the serum samples were serially diluted by 2-fold (ranging from 1:8 to 1:1024). These diluted serum samples were then exposed to a homologous virus (O PA2 or A YC) at a concentration of [100 × the tissue culture infectious dose (TCID)_50_]/0.5 mL and incubated for 1 h at 37°C. After 1 h, an LF-BK cell suspension was introduced to all wells. CPE was noted after 2–3 days in order to determine the titers, which were presented as the log_10_ of the reciprocal antibody dilution necessary to neutralize 100 × TCID_50_ of the virus (Fowler et al., 2010; Fukai et al., 2015a).

#### 2.10.3. Immunoglobulin subtype (IgG, IgA, and IgM) ELISA

To detect specific Ig isotype antibodies, we carried out ELISA targeting porcine IgG, IgA, and IgM (Bethyl Laboratories. Inc., Montgomery, Texas, USA) within the serum samples according to the manufacturer’s instructions. In brief, diluted serum and standards were introduced to their respective wells, each with a volume of 100 μL/well, and incubated for 1 h at RT. Afterward, the plates were rinsed and allowed to air-dry. Following this, 100 μL of the 1× biotinylated detection antibodies were treated into all wells, and the plates were subjected to incubation at RT for 1 h. Following rinsing and drying of the wells, 100 μL of 1× streptavidin-horseradish peroxidase conjugate was introduced into each well. Subsequently, the plates underwent a 30 min incubation at RT. Following this, the plates underwent an additional cycle of rinsing and drying. Peroxidase activity was visualized by adding 100 μL/well of 1× TMB solution and allowing the plates to incubate at RT for 30 min. The reaction was terminated with 100 μL of 2 N H_2_PO_4_, and the absorbances at 450 nm were detected using a Hidex 300SL spectrophotometer (Hidex).

### 2.11. RNA isolation, cDNA synthesis, and quantitative real-time PCR (qRT-PCR)

Isolated porcine PBMCs were employed for RNA extraction via a RNeasy Mini Kit (QIAGEN, Valencia, CA, USA) and TRIzol^®^ reagent (Invitrogen; Thermo Fisher Scientific, Inc., Carlsbad, CA, USA). Subsequently, reverse transcription into cDNA was accomplished using a GoScript Reverse Transcription System (Promega) according to the manufacturer’s guidelines. The reverse-transcribed cDNA were then subjected to qRT-PCR using a Bio-Rad CFX96^™^ Touch Real-time PCR system. Quantitative gene expression levels were normalized to that of HPRT (endogenous housekeeping gene) and presented relative to the control values. The list of primers used in this study is presented in Supplementary Table S1.

### 2.12. Statistical analysis

All quantitative data are expressed as the mean ± standard error (SEM) unless otherwise specified. Variations between groups were evaluated utilizing two-way ANOVA with Tukey’s *post hoc* test or one-way ANOVA followed by Tukey’s *post hoc* test or Dunnett’s *post hoc* test, as appropriate. Statistical significance is indicated as follows: ^∗^*p* < 0.05, ^∗∗^*p* < 0.01, ^∗∗∗^*p* < 0.001, and ^****^p < 0.0001. Parametric examinations were employed to compare diverse groups. Survival curves were generated using the Kaplan–Meier method, and variations were analyzed using log-rank sum tests. GraphPad Prism 10.0.2 (GraphPad, San Diego, CA, USA) was used for all statistical analyses.

## 3. Results

### 3.1. Glycyrrhizic acid can stimulate an innate immune response

Prior to performing the subsequent experiments using glycyrrhizic acid, we assessed glycyrrhizic acid-mediated cytotoxicity via an MTS assay. Glycyrrhizic acid at various concentration did not exhibit cytotoxicity on any of the cell lines used in the study, including BHK-21, LF-BK, and ZZ-R cells, murine PECs, and porcine PBMCs (Supplementary Figure S1).

**Figure 1:**
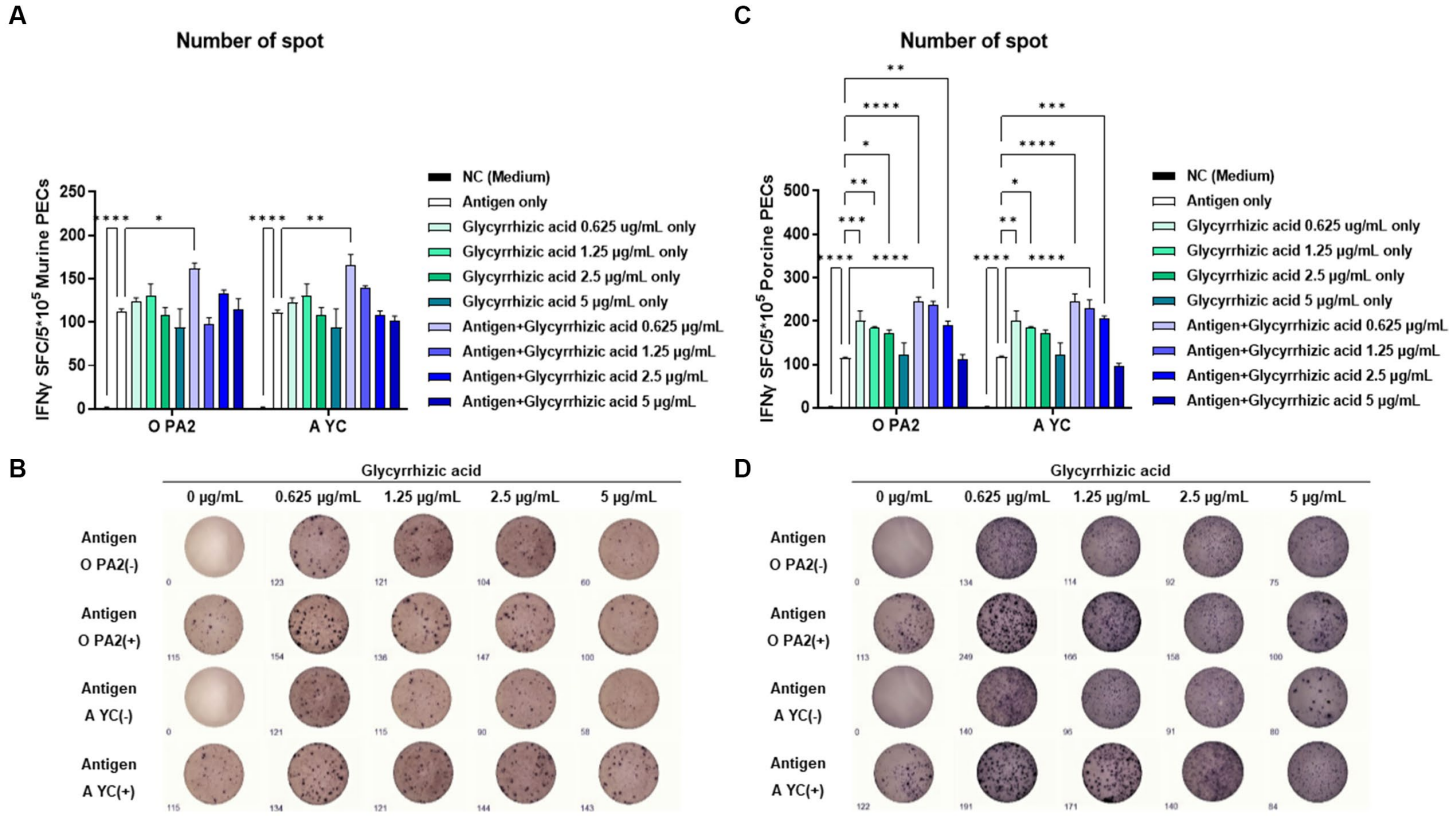
Inactivated FMDV type O (O PA2) or A (A YC) antigen-mediated innate immune response (in the presence or absence of glycyrrhizic acid) via secretion of interferon (IFN)γ in murine peritoneal exudate cells (PECs) and porcine peripheral blood mononuclear cells (PBMCs). To evaluate the innate immune response to inactivated FMDV type O (O PA2) or A (A YC) antigen (in the presence or absence of glycyrrhizic acid), IFNγ secretion was determined using an ELISpot assay. **(A–D)** IFNγ-secreting cell spots in murine PECs **(A)**; Images of IFNγ secretion in murine PECs **(B)**; IFNγ-secreting cell spots in porcine PBMCs **(C)**; Images of IFNγ secretion in porcine PBMCs **(D)**. Data are represented as the means ± SEM of spot-forming cells (SFCs) from triplicate measurements (*n* = 3/group). Statistical analyses were conducted using one-way ANOVA followed by Tukey’s *post-hoc* test. ^*^p < 0.05, ^**^p < 0.01, ^***^p < 0.001, and ^****^p < 0.0001.

To demonstrate the innate and cellular immune response-inducing potential of glycyrrhizic acid, we conducted IFNγ ELISpot analysis by including glycyrrhizic acid at varying concentrations with or without inactivated FMDV (O PA2 or A YC) antigens. This assay used murine PECs and porcine PBMCs. When murine PECs were treated with 0.625 μg/mL glycyrrhizic acid along with FMDV (O PA2 or A YC) antigens, a significant increase in IFNγ secretion was observed compared to when antigen alone was administered (Figures 1A,B). Furthermore, in porcine PBMCs, it was observed that, apart from glycyrrhizic acid at 5 μg/mL, all concentrations led to a significant increase in IFNγ secretion when compared to antigen only (Figures 1C,D). Thus, glycyrrhizic acid is able to stimulate innate and cellular immune responses.

### 3.2. Glycyrrhizic acid as an adjuvant induces a strong host defense against FMDV infection in mice

Prior to evaluating the FMD vaccine containing glycyrrhizic acid-mediated host defense, we investigated the efficacy of glycyrrhizic acid alone in inducing host defense responses against FMDV O/VET/2013 and A/Malay/97 infection in mice. In previous experiments, glycyrrhizic acid significantly increased IFNγ secretion via induction of an innate immune response. However, our results showed that glycyrrhizic acid alone (without inactivated FMDV antigen) did not induce a host defense response against viral infection (Supplementary Figures S2B–E).

**Figure 2:**
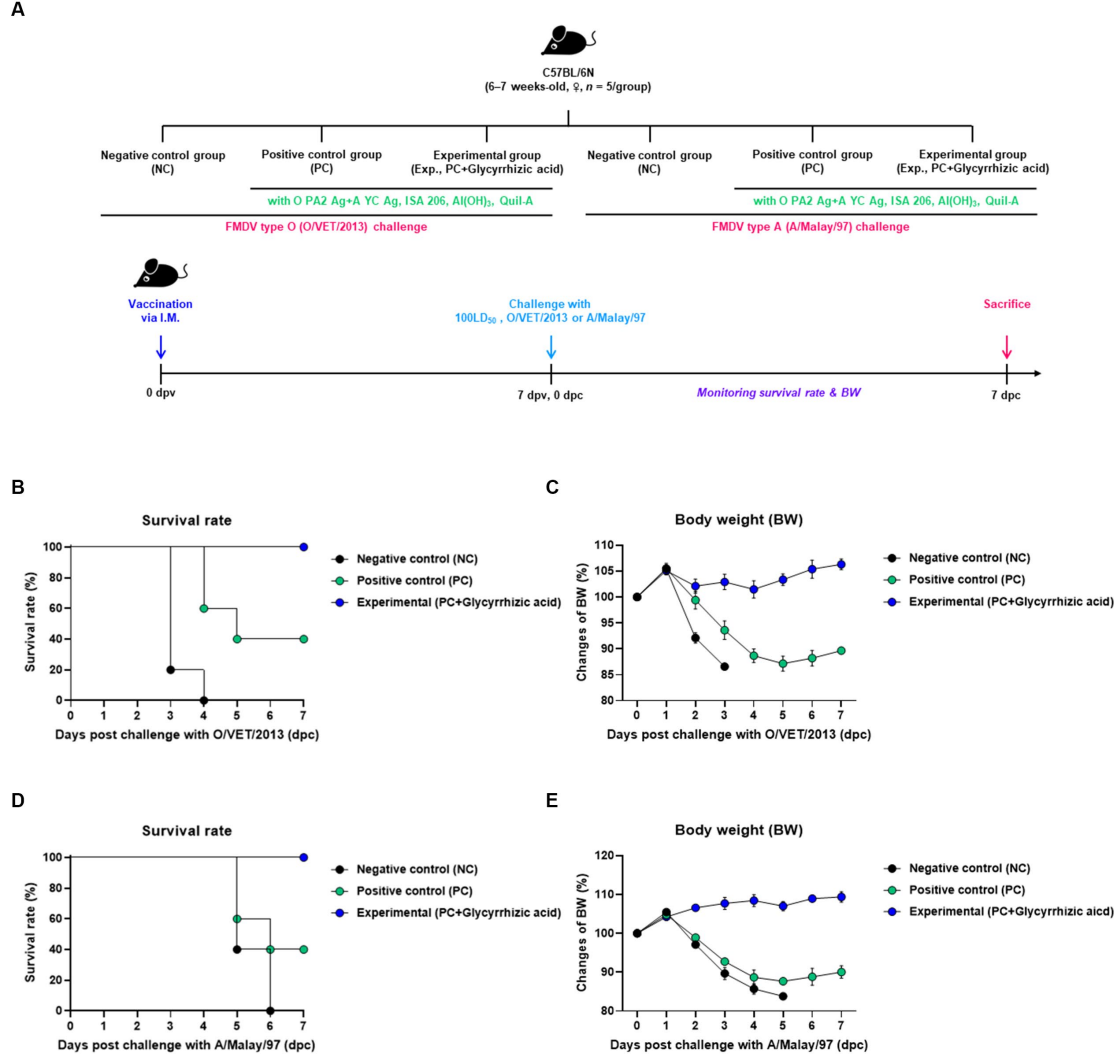
Efficacy and protective effects of FMD vaccine (containing glycyrrhizic acid) in the early stage of vaccination against viral infection in mice. C57BL/6 mice (females, 6–7 weeks old, *n* = 5/group) were divided into six groups, namely, a negative control (NC) group for FMDV type O (O/VET/2013) challenge (*n* = 5/group), a positive control (PC) group for FMDV type O (O/VET/2013) challenge (*n* = 5/group), an experimental (Exp.) group for FMDV type O (O/VET/2013) challenge (*n* = 5/group), an NC group for FMDV type A (A/Malay/97) challenge (*n* = 5/group), a PC group for FMDV type A (A/Malay/97) challenge (*n* = 5/group), and an Exp. group for FMDV type A (A/Malay/97) challenge (*n* = 5/group). The Exp. group was administered test vaccines containing 0.375 μg of O PA2 + 0.375 μg of A YC antigen (1/40 dose for cattle and pig use) with ISA 206 (oil-based emulsion, 50%, w/w), 10% Al(OH)_3_, 15 μg Quil-A, and 100 μg glycyrrhizic acid. The PC group was administered all the above except glycyrrhizic acid. The NC group was injected with an equivalent amount of PBS. Vaccination was performed once with 100 μL vaccine (1/10 the normal dose for cattle and pigs) injected intramuscularly into the thigh, and the mice were challenged with FMDV type O (100 LD_50_ O/VET/2013) and FMDV type A (100 LD_50_ A/Malay/97) at 7 days post-vaccination (dpv). Survival rates and body weights were monitored for 7 days post-challenge (dpc). **(A–E)** Experimental strategy **(A)**; Survival rates post-challenge with O/VET/2013 **(B)**; Changes in body weight post-challenge with O/VET/2013 **(C)**; Survival rates post-challenge with A/Malay/97 **(D)**; Changes in body weight post-challenge with A/Malay/97 **(E)**. Data are represented as the means ± SEM of triplicate measurements (*n* = 5/group).

In order to evaluate the protective effect of the test vaccine (containing glycyrrhizic acid as an adjuvant) against FMDV infection, the experiment was performed according to the protocol shown in Figure 2A. A 100% survival rate was seen in the group which was vaccinated with the glycyrrhizic acid adjuvant (Exp. Group) post infection with FMDV type O (O/VET/2013) compared to that in both the PBS-injected (NC) group (0% survival rate) and the test vaccine without glycyrrhizic acid-vaccinated (PC) group (40% survival rate) (Figure 2B). When experiments were conducted using FMDV type A (A/Malay/97), the survival rate was 100% in the Exp. group (Figure 2D). The NC and PC groups lost weight while the Exp. group showed an increasing trend in weight (Figures 2C,E).

### 3.3. Efficacy of vaccines containing glycyrrhizic acid in inducing early, mid-term, and long-term immunity in mice

**Figure 3:**
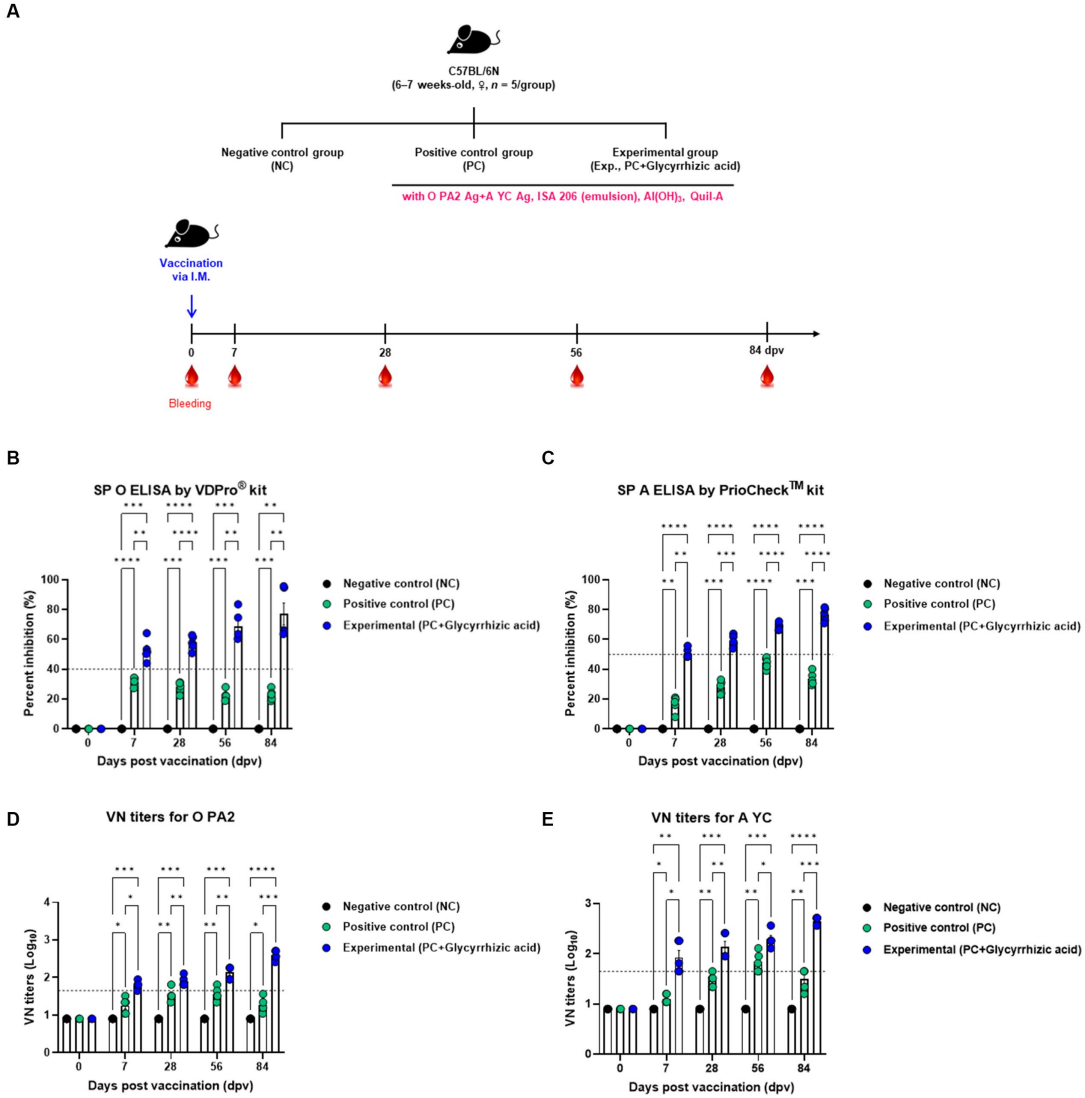
FMD vaccine containing glycyrrhizic acid mediates early, and mid- and long-term immune responses in mice. C57BL/6 mice (females, 6–7 weeks old, *n* = 5/group) were divided into three groups, namely, a negative control (NC) group (*n* = 5/group), a positive control (PC) group (*n* = 5/group), and an experimental (Exp.) group (*n* = 5/group). The Exp. group was administered test vaccines containing 0.375 μg of O PA2 + 0.375 μg of A YC antigen (1/40 dose for cattle and pig use) with ISA 206 (oil-based emulsion, 50%, w/w), 10% Al(OH)_3_, 15 μg Quil-A, and 100 μg glycyrrhizic acid in a total volume of 100 μL. The PC group was administered all the above except glycyrrhizic acid. The NC group was injected with an equivalent amount of PBS. Vaccination was performed once with 100 μL of vaccine (1/10 of the normal dose for cattle and pigs) injected intramuscularly into the thigh. Blood samples were collected at 0, 7, 28, 56, and 84 days post-vaccination (dpv) for serological assays. **(A–E)** Experimental strategy **(A)**; SP O antibody titers (VDPro^®^ kit) **(B)**; SP A antibody titers (PrioCheck^™^ kit) **(C)**; VN titers for O PA2 **(D)**; VN titers for A YC **(E)**. Data are represented as the means ± SEM of triplicate measurements (*n* = 5/group). Statistical analyses were conducted using two-way ANOVA followed by Tukey’s test. ^*^p < 0.05; ^**^p < 0.01; ^***^p < 0.001; and ^****^p < 0.0001.

In order to confirm that the test vaccine (with glycyrrhizic acid as an adjuvant) elicits an immune-enhancing effect as well as innate and adaptive (cellular and humoral) immune responses, we evaluated early, mid-, and long-term immunity in mice (Figure 3A). Mice were divided into three groups (NC, PC, and Exp.) and samples of blood were collected for serological analyses, which included SP O ELISA, SP A ELISA, and VN tests (Figures 3B–E).

After vaccination, antibody titers initially increased in both the PC and Exp. groups; however, the PC group’s antibody titers gradually decreased over time, while the Exp. group’s increased (measured by SP O ELISA and SP A ELISA) (Figures 3B,C). In terms of VN titers against O PA2 and A YC, the Exp. group demonstrated higher levels than the PC group, and this difference increased significantly with time (Figures 3D,E).

Therefore, the test vaccine, which includes glycyrrhizic acid as an adjuvant, demonstrated exceptional immunity-inducing effects in mice, including early (7 dpv), mid- (28 dpv), and long-term (56 and 84 dpv) immune responses.

### 3.4. Efficacy of vaccines containing glycyrrhizic acid in inducing early, mid-term, and long-term immunity in pigs

**Figure 4:**
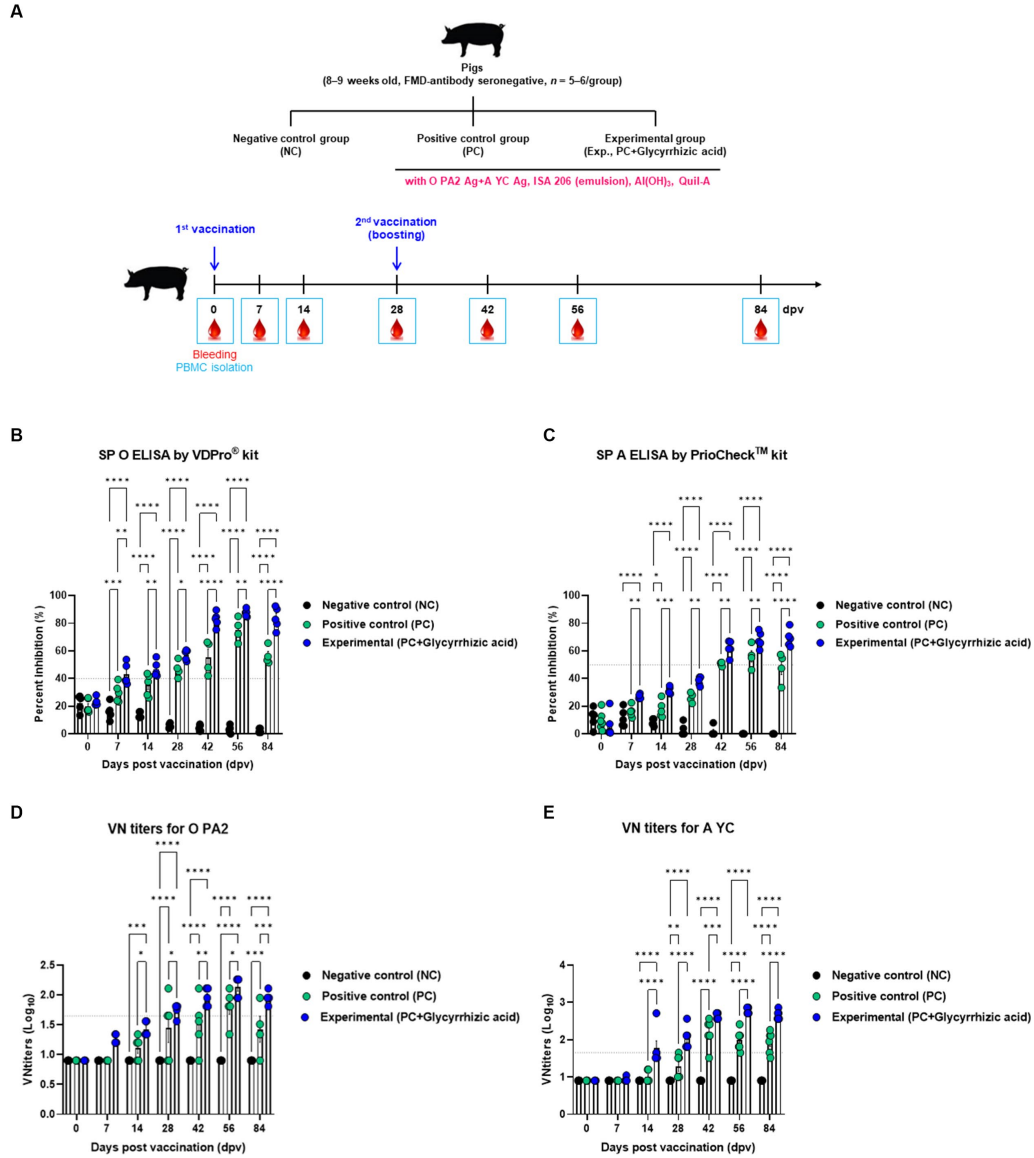
FMD vaccine containing glycyrrhizic acid mediates early, and mid- and long-term immune responses in pigs. Pigs (8–9 weeks old, FMD antibody-seronegative, *n* = 5–6) were divided into three groups, namely, a negative control (NC) group (*n* = 5–6/group), a positive control (PC) group (*n* = 5–6/group), and an experimental (Exp.) group (*n* = 5–6/group). The Exp. group was administered test vaccines containing 15 μg O PA2 + 15 μg A YC antigen (1 dose for cattle and pig use) with ISA 206 (oil-based emulsion, 50%, w/w), 10% Al(OH)_3_, 150 μg Quil-A, and 1 mg glycyrrhizic acid. The PC group was administered all the above except glycyrrhizic acid. Vaccination was performed twice at 28-day intervals, with 1 mL vaccine (1 dose for cattle and pigs) injected intramuscularly in the neck. Blood samples were collected from the pigs at 0, 7, 14, 28, 42, 56, and 84 days post-vaccination for serological assays. **(A–E)** Experimental strategy **(A)**; SP O antibody titers (VDPro^®^ kit) **(B)**; SP A antibody titers (PrioCheck^™^ kit) **(C)**; O PA2 VN titers **(D)**; A YC VN titers **(E)**. Data are represented as the means ± SEM of triplicate measurements (*n* = 5–6/group). Statistical analyses were conducted using two-way ANOVA followed by Tukey’s test. ^*^p < 0.05; ^**^p < 0.01; ^***^p < 0.001; and ^****^p < 0.0001.

Next, we conducted animal experiments on target animals to confirm whether glycyrrhizic acid as an adjuvant has synergistic effects in inducing innate and adaptive (cellular and humoral) immunity. FMD antibody-seronegative pigs were vaccinated with test vaccines (containing glycyrrhizic acid as an adjuvant), and cellular and humoral immune responses were evaluated along with early, mid-, and long-term immunity. A booster vaccine was administered at 28 dpv. Blood samples were collected at 0, 7, 14, 28, 42, 56, and 84 dpv, and serological tests were performed (Figure 4A).

**Figure 5:**
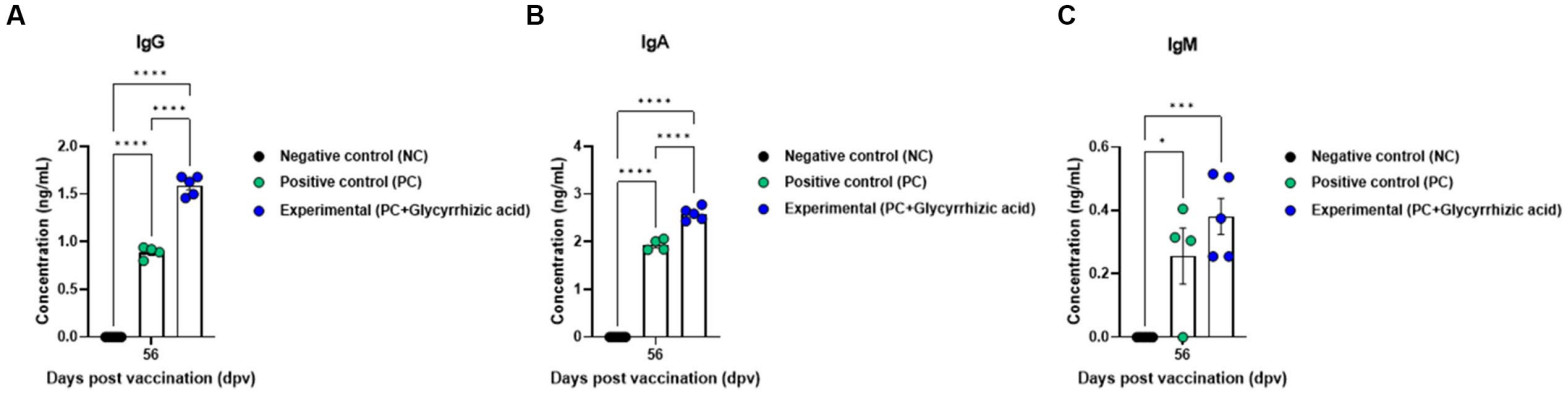
FMD vaccine containing glycyrrhizic acid mediates increases in immunoglobulin levels, including IgG, IgM, and IgA, in pigs. Pigs (8–9 weeks old, FMD antibody-seronegative, *n* = 5–6) were divided into three groups, namely, a negative control (NC) group (*n* = 5–6/group), a positive control (PC) group (*n* = 5–6/group), and an experimental (Exp.) group (*n* = 5–6/group). The Exp. group was administered test vaccines containing 15 μg O PA2 + 15 μg A YC antigen (1 dose for cattle and pig use) with ISA 206 (oil-based emulsion, 50%, w/w), 10% Al(OH)_3_, 150 μg Quil-A, and 1 mg glycyrrhizic acid. The PC group was administered all the above except glycyrrhizic acid. Vaccination was performed twice at 28-day intervals, with 1 mL vaccine (1 dose for cattle and pigs) injected intramuscularly in the neck. Blood samples from pigs were collected at 0, 7, 14, 28, 42, 56, and 84 days post-vaccination for serological assays. **(A–C)** IgG concentration **(A)**; IgA concentration **(B)**; IgM concentration **(C)**. Data are represented as the means ± SEM of triplicate measurements (*n* = 5–6/group). Statistical analyses were conducted using two-way ANOVA followed by Tukey’s test. ^*^p < 0.05; ^**^p < 0.01; ^***^p < 0.001; and ^****^p < 0.0001.

Results showed that after the initial vaccination period, the Exp. group had significantly higher antibody titers (measured via SP O ELISA and SP A ELISA) than the PC group, and at 84 dpv, antibody titers in the PC group had decreased, whereas those in the Exp. group remained consistent or increased (Figures 4B,C). Similarly, VN titers for O PA2 and A YC showed comparable results based on SP O ELISA and SP A ELISA (Figures 4D,E). Furthermore, an immunoassay was performed using sera from vaccinated pigs to assess the impact of vaccination (with a glycyrrhizic acid as an adjuvant) on immunoglobulin levels (Figures 5A–C). At 56 dpv, the concentrations of both IgG and IgA were significantly higher in the Exp. group than in the PC group (Figures 5A,B). In terms of IgM levels, inter-individual differences were observed but the overall level of IgM was elevated in the Exp. group when compared with the PC group at 56 dpv (Figure 5C).

Overall, the test vaccine containing glycyrrhizic acid showed superior immune-enhancing effects in pigs when compared to the vaccine without glycyrrhizic acid in terms of early- (7 and 14 dpv), mid- (28 and 42 dpv), and long-term (56 and 84 dpv) immunity.

### 3.5. FMD vaccine with glycyrrhizic acid adjuvant induces strong cellular and adaptive immune responses by inducing pattern-recognition receptor (PRR), transcription factor, cytokine, and co-stimulatory molecule expression in PBMCs

**Figure 6:**
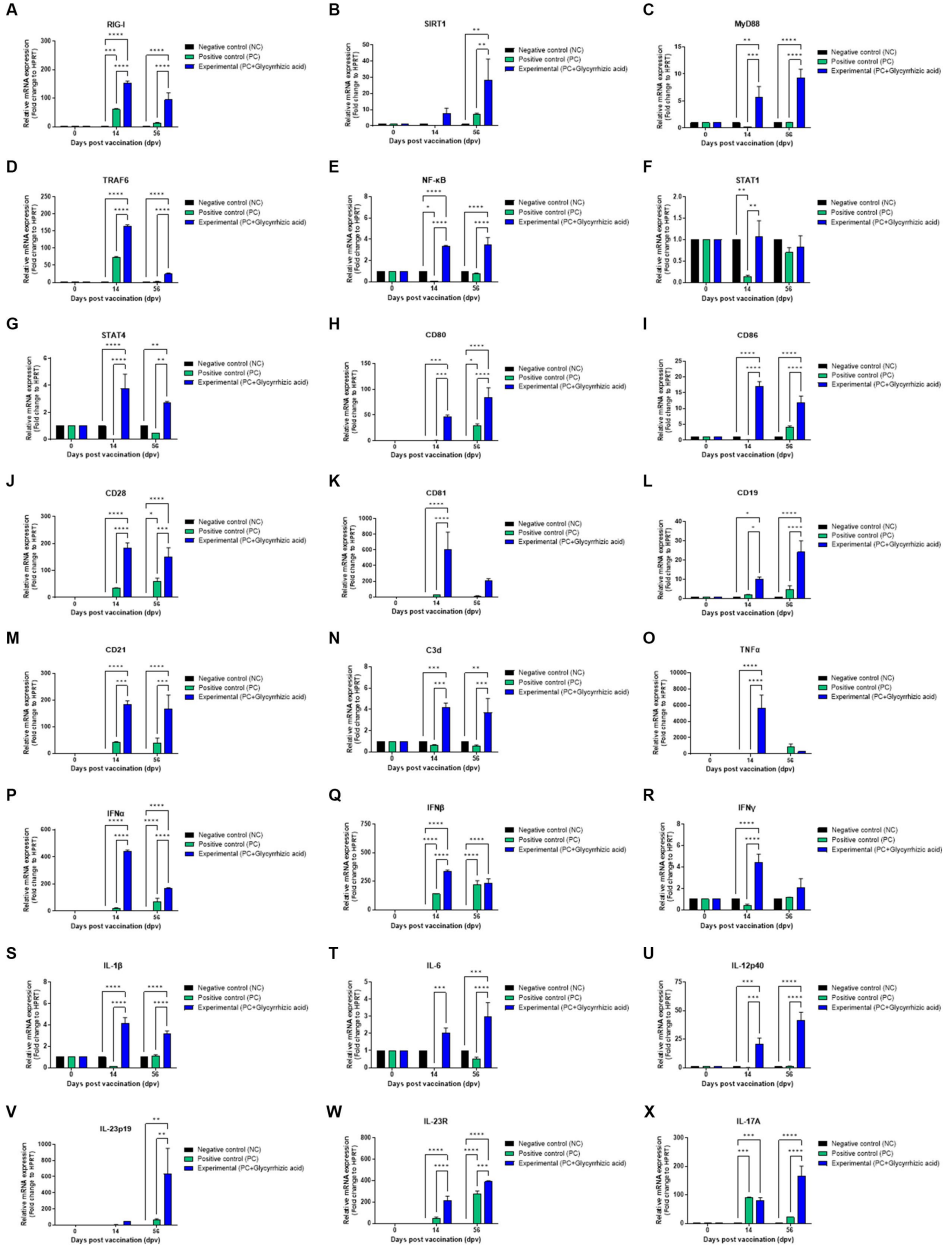
FMD vaccine containing glycyrrhizic acid mediates immunoregulatory gene expression in porcine peripheral blood mononuclear cells isolated from vaccinated pigs. Porcine peripheral blood mononuclear cells (PBMCs) isolated from the whole blood of vaccinated pigs [*n* = 5–6/group; as described in Figure 4 **(A)**] were used for qRT-PCR assays. Gene expression levels were normalized to HPRT levels and presented as a relative ratio to the control. **(A–X)** Gene expression levels of RIG-I **(A)**; SIRT1 **(B)**; MyD88 **(C)**; TRAF6 **(D)**; NF-κB **(E)**; STAT1 **(F)**; STAT4 **(G)**; CD80 **(H)**; CD86 **(I)**; CD28 **(J)**; CD81 **(K)**; CD19 **(L)**; CD21 **(M)**; C3d **(N)**; TNFα **(O)**; IFNα **(P)**; IFNβ **(Q)**; IFNγ **(R)**; IL-1β **(S)**, IL-6 **(T)**; IL-12p40 **(U)**; IL-23p19 **(V)**; IL-23R **(W)**; and IL-17A **(X)**. Data are represented as the means ± SEM of triplicate measurements (*n* = 5–6/group). Statistical analyses were conducted using two-way ANOVA followed by Tukey’s test. ^*^p < 0.05; ^**^p < 0.01; ^***^p < 0.001; and ^****^p < 0.0001.

To determine the effectiveness and mechanisms of vaccines containing glycyrrhizic acid, we performed qRT-PCR using porcine PBMCs isolated from whole blood samples from pigs vaccinated with the test vaccine containing glycyrrhizic acid (Figures 6A–X). In the Exp. group, retinoic acid-inducible gene (RIG)-I expression had increased significantly at 14 dpv and decreased slightly at 56 dpv. However, these differences were also observed in the PC group (Figure 6A). In addition, Sirtuin (SIRT)1 expression significantly increased at 56 dpv (Figure 6B). Myeloid differentiation primary response (MyD)88 expression significantly increased over time (Figure 6C). Tumor necrosis factor (TNF) receptor associated factor (TRAF)6 increased significantly higher in the Exp. group than in the PC group at 14 dpv; however, levels in both groups decreased at 56 dpv, although they remained higher in the Exp. group (Figure 6D). Nuclear factor kappa-light-chain-enhancer of activated B cells (NF-κB) was also found to increase at both 14 dpv and 56 dpv (Figure 6E). Although signal transducer and activator of transcription (STAT)1 level had increased in the Exp. group compared to that in the PC group at 14 dpv, there was no difference at 56 dpv (Figure 6F). STAT4 expression increased significantly in the Exp. group compared to that in the PC group at 14 dpv and decreased slightly at 56 dpv, although it remained significantly higher in the PC group (Figure 6G).

Cluster of differentiation (CD)80, CD86, and CD28 levels were all significantly increased in the Exp. group compared to those in the PC group at both 14 and 56 dpv (Figures 6H–J). CD81 expression was significantly higher than that of PC group at 14 dpv; however, level in Exp. group decreased at 56 dpv, although they remained higher in the Exp. group (Figure 6K). Regarding CD19, CD21, and C3d levels, the Exp. group showed increases at 14 dpv and slightly higher levels at 56 dpv (Figures 6L–N). This indicated that glycyrrhizic acid is involved in the activation and proliferation of T and B cells by acting as a co-stimulating signal.

TNFα levels were significantly elevated in the Exp. group when compared with the PC group at 14 dpv (Figure 6O). IFNα, IFNβ, and IFNγ levels were significantly increased at 14 dpv; at 56 dpv, they were still higher than in the PC group, although they decreased thereafter (Figures 6P–R). Based on these results, glycyrrhizic acid appears to induce a balanced innate immune response.

Interleukin (IL)-1β and IL-6 levels significantly increased at 14 dpv and decreased at 56 dpv, but remained significantly higher in the Exp. group than in the PC group (Figures 6S,T). IL-12p40 levels increased over time and were significantly higher in the Exp. group (Figure 6U). IL-23p19 and IL-23R levels were also higher in the Exp. group when compared to the NC and PC groups at 56 dpv (Figures 6V,W).

No significant difference was observed in IL-17A levels between the PC and Exp. groups at 14 dpv; however, the level in the Exp. group was remarkably higher at 56 dpv (Figure 6X).

Collectively, these findings suggest that glycyrrhizic acid could stimulate RIG-I to induce inflammation, activate NF-κB through the MyD88 and TRAF6 signaling pathways despite SIRT1 differentiation, mediate responses to IL-12p40 via STAT4, and regulate the differentiation of T helper cells.

## 4. Discussion

FMD is a disease that affects hooved livestock and has been prevalent throughout history (Grubman and Baxt, 2004; Jamal and Belsham, 2013). In some European countries, emergency vaccination is mandatory once a FMD outbreak occurs, even if the country was considered FMD-free previously (Arzt et al., 2011; Stenfeldt et al., 2014).

Since its discovery, there have been many studies on vaccines for FMDV. Recombinant protein vaccines such as virus-like particle (VLP) vaccines (Xiao et al., 2016), peptide vaccines (Cao et al., 2013), and DNA (Kotla et al., 2016) and RNA (Borrego et al., 2017) vaccines have become a research hotspot. Alternatively, inactivated vaccines have the highest immunogenicity; however, shielding facilities (such as BSL3 facilities) are required to make inactivated vaccines and there is always a risk of live virus leakage due to incomplete inactivation. Therefore, currently commercially available vaccines are prepared in the form of double oil emulsions, where the oil-soluble adjuvant is mixed with the inactivated whole virus (Nagendrakumar et al., 2011).

Typically, an adjuvant is a substance that is administered with a vaccine and can enhance its efficacy by triggering a host immune response. Currently commercially available inactivated vaccines contain oil-based adjuvants. However, these vaccines have disadvantages including the long period required for antibody titers to rise to defensive levels post-vaccination, limiting their ability to induce an initial host response post-vaccination. Moreover, significant side effects (such as local adverse reactions at the vaccination site) can be caused by oil-based adjuvants.

Aluminum has been used as a safe adjuvant in many vaccines since 1926 (Ramon, 1925; Hogenesch, 2013), and is still widely used. However, Al(OH)_3_, the most commonly used adjuvant, has the limitation of only stimulating Th2-mediated immune responses (Hem and Hogenesch, 2007; Jiang et al., 2018).

If these limitations are overcome through the addition of novel immunostimulants, the immune response after vaccination can be expected to increase significantly. This may improve the efficacy of the vaccine in inducing immunity at all stages, including early, mid-term, and long-term immunity.

Therefore, we aimed to identify an immunostimulant without the side effects of oil-based adjuvants with broad immunogenic properties [as opposed to Al(OH)_3_, which only induces a Th2 immune response]. In this study, we developed an FMD vaccine which has potential to overcome the limitations of commercially available FMD vaccines.

An ideal adjuvant must be easy to obtain and handle, safe, and stable, and should be able to maximize vaccine effectiveness by enhancing host immunity. Plant extracts have been actively studied over the past 30 years as vaccine adjuvants, as they contain natural substances with medicinal properties and are easy to obtain (Surh, 2002). In addition, natural compounds from plant extracts have the advantages of being stable and safe in terms of molecular structure, making them suitable as adjuvants, and are hence widely used in the pharmaceutical fields.

Saponins have various effects including antioxidant, antiviral, ulcer prevention, and antibacterial effects; some are currently approved as food products and are used in medicines and cosmetics (Bailly and Vergoten, 2020). Quil-A extracted from the bark of *Quillaja saponaria* is currently used as a vaccine adjuvant (i.e., in HIV vaccines) and belongs to the triterpenoid family of saponins. However, Quil-A has serious drawbacks; it is highly toxic, unstable in liquid conditions, and demonstrates hemolytic effects. For these reasons, many studies have recently been conducted on the use of different natural saponins as immunostimulants (Marciani et al., 2003; Sun et al., 2009). Glycyrrhizic acid is a triterpenoid saponin extracted from licorice roots, a medicinal plant that has been used for approximately 4,000 years in both the East and the West. There are 22.2 to 32.3 g of glycyrrhizic acid per 100 g of dried licorice root. Glycyrrhizic acid is a potent immunoreactive anti-inflammatory agent that functions at the cellular and membrane levels. At the membrane level, it induces cholesterol-dependent degradation of lipid rafts which are important for coronavirus entry into cells. It traps high mobility group box 1 protein (HMGB1) at both intracellular and circulatory sites and inhibits the alarmin function of HMGB1. It also shows simultaneous antiviral and antitumor activity in clinical studies, and is known for its immunomodulatory function in dendritic cells via the expression of CD40, CD86, and major histocompatibility complex (MHC)-II markers (Bailly and Vergoten, 2020; Kenubih, 2021). Glycyrrhizic acid is suitable for use as a vaccine adjuvant based on its immunomodulatory properties, and licorice, the raw material, is readily available. However, the effectiveness of glycyrrhizic acid on inducing immune responses as an FMD vaccine adjuvant has not been studied previously.

A prior cell viability assay was conducted to explore whether various concentrations of glycyrrhizic acid are cytotoxic. No cytotoxicity was observed at the concentrations of glycyrrhizic acid used in our experiments (Supplementary Figure S1). We used ELISpot to investigate whether glycyrrhizic acid could promote innate immune cell-derived IFNγ expression and lead to innate immune and Th1 responses, with the aim of addressing the shortcomings of Al(OH)_3_, a currently used vaccine adjuvant (Figure 1).

Innate immune cells, including natural killer (NK) cells, identify and eliminate infected cells (which exhibit decreased MHC class I molecule expression) while also triggering the production of cytokines like IFNγ, IL-12, and IL-18. The secreted IFNγ further enhances the immune response by binding to NK cells, while cytokines such as IL-12 and IL-18 promote the differentiation of naïve CD4^+^ T cells into Th1 cells (Annunziato et al., 2015). When glycyrrhizic acid was administered at a concentration of 0.625 μg/mL alongside the studied viral antigens (O PA2 and A YC), IFNγ expression in murine PECs and porcine PBMCs was maximized (Figure 1). The results suggest that glycyrrhizic acid can sufficiently stimulate the innate immune system to promote IFNγ expression and induce Th1 responses even at low concentrations, thereby overcoming the shortcomings of Al(OH)_3_ adjuvants which only induce a Th2 response.

Since the peritoneal cavity contains many naive macrophages, it is a preferred site for the collection of naive tissue-resident macrophages. We have described PECs and discussed the reasons for using them in experiments in previous studies (Jo et al., 2021; Kim et al., 2023). PECs include innate immune cells such as antigen-presenting cells (APCs), which include dendritic cells, macrophages, monocytes, and unconventional T cells. Therefore, PECs are especially suitable for studying cellular and systemic immune responses.

Bailly and Vergoten (2020) reported the antiviral effects of glycyrrhizic acid against various human viruses including hepatitis A/B/C virus, Epstein Barr virus (EBV), Dengue virus (DENV), Chikungunya virus (CHIKV), Semliki Forest virus (SFV), parainfluenza virus, Varicella-zoster virus (VZV), and influenza virus. In particular, glycyrrhizic acid is known to have potent anti-HIV1 and anti-SARS-CoV-2 activities. Glycyrrhizic acid also has antiviral activity against several animal viruses, such as duck hepatitis virus (DHV), avian infectious bronchitis virus (IBV), porcine reproductive and respiratory syndrome virus (PRRSV), and porcine epidemic diarrhea virus (PEDV).

Therefore, before evaluating the adjuvanticity of glycyrrhizic acid, we first performed mice experiments to determine whether glycyrrhizic acid alone exerts a protective effect in the host against FMDV infection. However, our results showed that glycyrrhizic acid alone did not protect the host against viral infection (Supplementary Figure S2).

Next, animal experiments were performed using experimental (mice) and target (pigs) animals to evaluate the adjuvanticity (immunostimulatory effect) of glycyrrhizic acid. A vaccine containing O PA2 and A YC antigens, ISA 206, Al(OH)_3_, and Quil-A was administered to the PC group. Glycyrrhizic acid was added to the vaccine administered to the Exp. group. To verify that the vaccine containing glycyrrhizic acid was effective in inducing an early defense response, animal experiments were conducted using mice to assess their survival rate and body weight; the Exp. group exhibited a higher survival rate and increased body weight when compared to the PC group (Figure 2). These results suggest that glycyrrhizic acid has sufficient effects on early defense responses when used as an adjuvant.

Subsequently, animal experiments using mice and pigs were conducted to evaluate whether vaccines containing glycyrrhizic acid have a positive effect on early-, mid-, and long-term immunity. In mice, antibody titers increased at 7 dpv in the PC group while they increased steadily until 84 dpv in the Exp. group (Figure 3). In the target animal experiment, antibody titers were confirmed to increase faster in the Exp. group than in the PC group after boosters were administered (Figure 4).

In the case of the Exp. group, the inter-individual difference was smaller than that of the PC group. A critical issue in pigs is that that complete host defense is difficult due to the large inter-individual differences in antibody and VN titers after vaccination. Thus, glycyrrhizic acid may improve resistance against FMDV by inducing more consistent immunity across individuals.

In addition, VN titers for O PA2 and A YC were measured to confirm the virus neutralization effects of the vaccine. In the mice experiment, only one individual in the PC group had a VN titer greater than 1.65 Log_10_ at 28 dpv, and titers in all individuals had dropped by 84 dpv. In contrast, most of the individuals in the Exp. group had titers of 1.65 Log_10_ or higher at 7 dpv, and the titers had increased further by 84 dpv (Figure 3). When assessing VN titers in the target animals, individuals in the Exp. group had superior titers over those in the PC group (Figure 4). Regarding A YC, the Exp. group demonstrated VN titers of 1.65 Log_10_ or greater at 14 dpv, and maintained this increase at 84 dpv. When considering O PA2, although both the Exp. and PC groups demonstrated a reduction in VN titers at 84 dpv, the Exp. group consistently demonstrated levels of 1.65 Log_10_ or higher.

Previous studies have suggested that FMD vaccine-induced serum titers higher than 1.74 Log_10_ in pigs can be used as an alternative to challenge experiments with oil emulsion vaccines (Black et al., 1984). As per the evaluation standards for FMD vaccines in Korea (Jo et al., 2021), a VN titer of 1.65 Log_10_ or higher post-vaccination indicates that the host is capable of defending against viral challenge. According to the WOAH guidelines, it is recommended to challenge pigs 4 weeks (28 days) post-vaccination to evaluate host defenses against viral infection. In this study, the FMD vaccine containing glycyrrhizic acid as an adjuvant-induced VN titer higher than 1.65 Log_10_ for both FMDV types O and A at 28 dpv, whereas the PC group (FMD vaccine without glycyrrhizic acid) showed VN titers lower than 1.65 Log_10_. Based on these results, although we did not conduct FMDV challenge experiments in this study, we speculated that an FMD vaccine containing glycyrrhizic acid would exhibit effective host protection against viral infection in pigs.

Vaccines containing glycyrrhizic acid had superior efficacy in inducing early-, mid-, and long-term immune responses, as represented by increased levels of IgG, IgA, and IgM. IgM is the first immunoglobulin to be generated during infection, and its levels increase in the blood to counter external intrusion. Following that, IgG levels rise, and IgM and IgG activate complement to induce an immune response against the invading pathogen. IgA is mainly secreted from the mucous membranes to defend the mucosa. IgG and IgA levels were higher in the Exp. group than in the PC group; IgM levels were also higher in the Exp. group (on an average), although this difference was not statistically significant. Based on these results, vaccines with glycyrrhizic acid as an adjuvant can efficiently elicit active immunity by inducing cellular immune responses shortly after vaccination, stimulating humoral immune responses, and maintaining these responses long-term (Figure 5).

We performed qRT-PCR to elucidate the mechanisms of how the test bivalent vaccine (containing glycyrrhizic acid as an adjuvant) elicited innate and adaptive (cellular and humoral) immune responses (Figure 6).

The expression of RIG-I, a member of the RIG-I-like receptor (RLR) family, was significantly increased in the Exp. group when compared with the PC group. RIG-I plays a crucial role in initiating the immune response by detecting and identifying viral RNA, leading to the activation of the type I interferon (IFNα and IFNβ) response. Furthermore, at 14 dpv, there was a significant increase in the levels of type I interferons and TRAF6. At 56 dpv, we observed that IFNβ expression had decreased in the Exp. group when compared to 14 dpv but remained higher than that of the PC group. Additionally, both IFNα and TRAF6 levels remained significantly elevated when compared to the PC group. In summary, glycyrrhizic acid has been confirmed to exert a positive influence on cellular immune responses when used as an adjuvant.

IL-12 plays a pivotal role in promoting the differentiation of naïve T cells toward Th1 cells (Hsieh et al., 1993). This is instrumental in inducing IFNγ and TNFα production, forming a critical bridge between innate and adaptive immunity. IL-12Rβ1 and IL-12Rβ2, via phosphorylation of STAT4, help stimulate the expression of IFNγ. IL-23 is a heterodimeric cytokine that comprises the IL-12p40 and IL-23p19 subunits and belongs to the IL-12 family (Schön and Erpenbeck, 2018). IL-23 is vital in amplifying and sustaining Th17 cells and unconventional T cells. When it binds to IL-1β, it triggers the expression of IL-17. Notably, IL-17A recruit neutrophils to the site of pathogenic infection, where it forms neutrophil extracellular traps (NETs). This process is essential in early-stage host defenses against infections (Papayannopoulos, 2018; Giusti et al., 2019). IL-1β, IL-12p40, IL-23p19, IL-23R, and IL-17A levels were confirmed to gradually increase over a long period post-vaccination (56 dpv) in the Exp. group. Further, STAT4 levels slightly decreased at 56 dpv, but remained significantly higher than that in the PC group. These results suggest that glycyrrhizic acid affects early host defense and can simultaneously induce innate and adaptive immune responses by enhancing IFNγ expression and Th1 cell stimulation.

The Exp. group showed a significant increase in type II IFN (IFNγ) expression in the mid-term post-vaccination (14 dpv), and retained higher IFNγ expression than the PC group in the long-term (56 dpv). IFNγ is an important autocrine signal for APCs and can cause T cell-mediated cellular immune responses. In addition, STAT1 levels increased significantly at 14 dpv when compared to that in the PC group. STAT1 responds to IFNγ stimulation by forming a heterodimer with STAT2 which binds to the Interferon-Stimulated Response Element (ISRE) promoter, thereby affecting immune regulation. Collectively, glycyrrhizic acid was confirmed to increase the expression of both type I and type II IFNs, thereby increasing the immune response.

MyD88 is an immunoregulatory gene that plays an important role in innate immunity. It mediates the triggering of inflammatory cytokines through NF-κB via TRAF6 (Sugiyama et al., 2016) and affects immune-related membrane proteins such as CD28, CD80, and CD86. The detached NF-κB translocates to the nucleus, where it attaches to a distinct DNA sequence and promotes cytokine release. MyD88 levels were found to decrease over time in the PC group, whereas they increased significantly over time in the Exp. group. NF-κB levels increased significantly from 14 dpv in the Exp. group, and this increase was maintained at 56 dpv. This increase could be attributed to SIRT1. SIRT1 is known to suppress NF-κB by inactivating p53 (Gonfloni et al., 2014). SIRT1 levels had increased significantly in the Exp. group at 56 dpv. These results suggest that glycyrrhizic acid, by regulating the expression of NF-κB, can consistently provide immune stimulation without triggering a cytokine storm, and its use as an adjuvant may enhance the safety of vaccines.

Collectively, we propose a putative mechanism of how the FMD vaccine (containing glycyrrhizic acid) induces an immune response in pigs as follows; first, after vaccination with the test vaccine containing glycyrrhizic acid, glycyrrhizic acid-antigen complexes become bound to porcine PBMCs and are endocytosed. RIG-I is stimulated after recognition of the glycyrrhizic acid-antigen complexes, inducing NF-κB activation through TRAF6 expression. SIRT1 suppresses excessive NF-κB activity, thereby suppressing the inflammatory response and maintaining homeostasis in the host. NF-κB promotes the secretion of proinflammatory cytokines (such as IL-1β, IFNα, IFNβ, IFNγ, TNFα, IL-12p40, and IL-23p19) via activation of inflammasomes, and the secreted cytokines enter cells through their respective receptors (such as IL-1β, IL-12R, and IL-23R). These receptors are present on the surface of APCs, conventional T cells, and unconventional T cells, and these interactions induce a reversible response. The induced responses lead to cytokine (i.e., NF-κB and IFNγ) release from APCs, conventional T cells, and unconventional T cells via MyD88, STAT1, and STAT4. Specifically, IL-17A (secreted from unconventional T cells) forms NETs to clear the virus in the early stages of viral infection, thereby protecting the host. In the mid-term immune response, CD80/86 (expressed on APCs) reacts with CD28 on the surface of CD4^+^ T cells to induce a cellular immune response, and IFNγ (secreted from conventional and unconventional T cells) stimulates naïve B cells to promote their differentiation into mature B cells. In the long-term immune response, glycyrrhizic acid promotes the expression of CD19-CD21-CD81 (a core receptor for B cells) and C3d (which acts as an adjuvant when binding an antigen), which also promotes the differentiation of memory B cells and induces a long-lasting immune response.

In summary, it has been proven that the test vaccine containing glycyrrhizic acid as an adjuvant induces an innate immune response, strengthens the initial defense mechanism, and induces improved antibody titers, VN titers, and immune-related gene expression in the long term. Consequently, glycyrrhizic acid has been shown to positively affect host defenses by contributing to cellular and humoral immune responses and inducing long-lasting immunity. Glycyrrhizic acid is a valuable adjuvant candidate as it is both soluble and economical, and also has potential as a practical novel immunomodulator because it specifically targets immune cell-mediated responses.

Nevertheless, the absence of challenge experiment to evaluate the host protective effect of FMD vaccines containing glycyrrhizic acid against viral infection is a critical limitation of this study. Demonstrating the effectiveness of host defense through viral challenge experiments is undoubtedly the most important factor in evaluating the efficacy of vaccines and adjuvants. FMDV challenge experiments in experimental (mice) and target (pigs) animals can only be conducted within ABSL3 facilities. Our institute is affiliated with the Korean government and is the only facility in Korea capable of conducting large scale of animal testing within an ABSL3. Due to the high demand for challenge experiment against veterinary viral disease including FMD and African swine fever (ASF) experiments and limited space, ABSL3 animal experiments are strictly managed on an annual plan. For this reason, we were unable to perform the challenge experiment in this study in pigs. Further studies are planned to evaluate the efficacy of the FMD vaccine, including assessments of the glycyrrhizic acid-mediated host defense response (based on clinical indicators, viral titers in sera and oral swab samples, etc.) in pigs.

Although this study demonstrated the effectiveness of glycyrrhizic acid as an adjuvant for FMD vaccine, further studies are needed for other diseases such as ASF virus (ASFV) and PRRSV.

The current study evaluated the immunomodulatory effects of glycyrrhizic acid when administered via the intramuscular route. Previous studies have shown that glycyrrhizic acid can be detected in human plasma after oral ingestion, and may have the potential to induce mucosal and systemic immune responses when administered orally (Suzuki et al., 2017). Oral administration offers advantages over direct intramuscular vaccination because it can simultaneously stimulate mucosal and systemic immunity, is convenient, and allows for the delivery of large doses (Brownlie, 1985; Wang et al., 2015).

Based on the current results, we are planning a future study to evaluate the feasibility and efficacy of glycyrrhizic acid in inducing immune responses when orally administered as a feed or drinking water additive. In addition, we aim to examine the potential utility of glycyrrhizic acid as a bait vaccine, especially for animal diseases that are difficult-to-prevent and control.

## Data availability statement

The original contributions presented in the study are included in the article/Supplementary material, further inquiries can be directed to the corresponding author.

## Ethics statement

The animal study was approved by the Ethics Committee of the APQA (accreditation number: IACUC-2022-670 and IACUC-2023-753). The study was conducted in accordance with the local legislation and institutional requirements.

## Author contributions

SS: Investigation, Validation, Writing – original draft. HWK: Formal analysis, Investigation, Visualization, Writing – original draft, Writing – review & editing. M-KK: Investigation, Writing – original draft. SHP: Investigation, Writing – original draft. S-MK: Resources, Writing – review & editing. J-HP: Resources, Writing – review & editing. MJL: Conceptualization, Data curation, Formal analysis, Funding acquisition, Investigation, Methodology, Project administration, Resources, Software, Supervision, Validation, Visualization, Writing – original draft, Writing – review & editing.
