# Supplementary material for: Inactivated vaccine with glycyrrhizic acid adjuvant elicits potent innate and adaptive immune responses against foot-and-mouth disease
Source: Front Microbiol. 2023 Oct 31;14:1289065. doi: 10.3389/fmicb.2023.1289065 (PMC10644816; doi:10.3389/fmicb.2023.1289065)
Supplement: Supplementary file 1 [file Data_Sheet_1.PDF]

Supplementary Material

Inactivated Vaccine with Glycyrrhizic Acid Adjuvant Elicits Potent Innate and Adaptive Immune Responses Against Foot-and-Mouth Disease

Seokwon Shin†, Hyeong Won Kim†, Mi-Kyeong Ko, So Hui Park, Su-Mi Kim, Jong-Hyeon Park, Min Ja Lee*

*** Correspondence:** Min Ja Lee: herb12@korea.kr

1 Supplementary Figures and Tables

1.1 Supplementary Figure

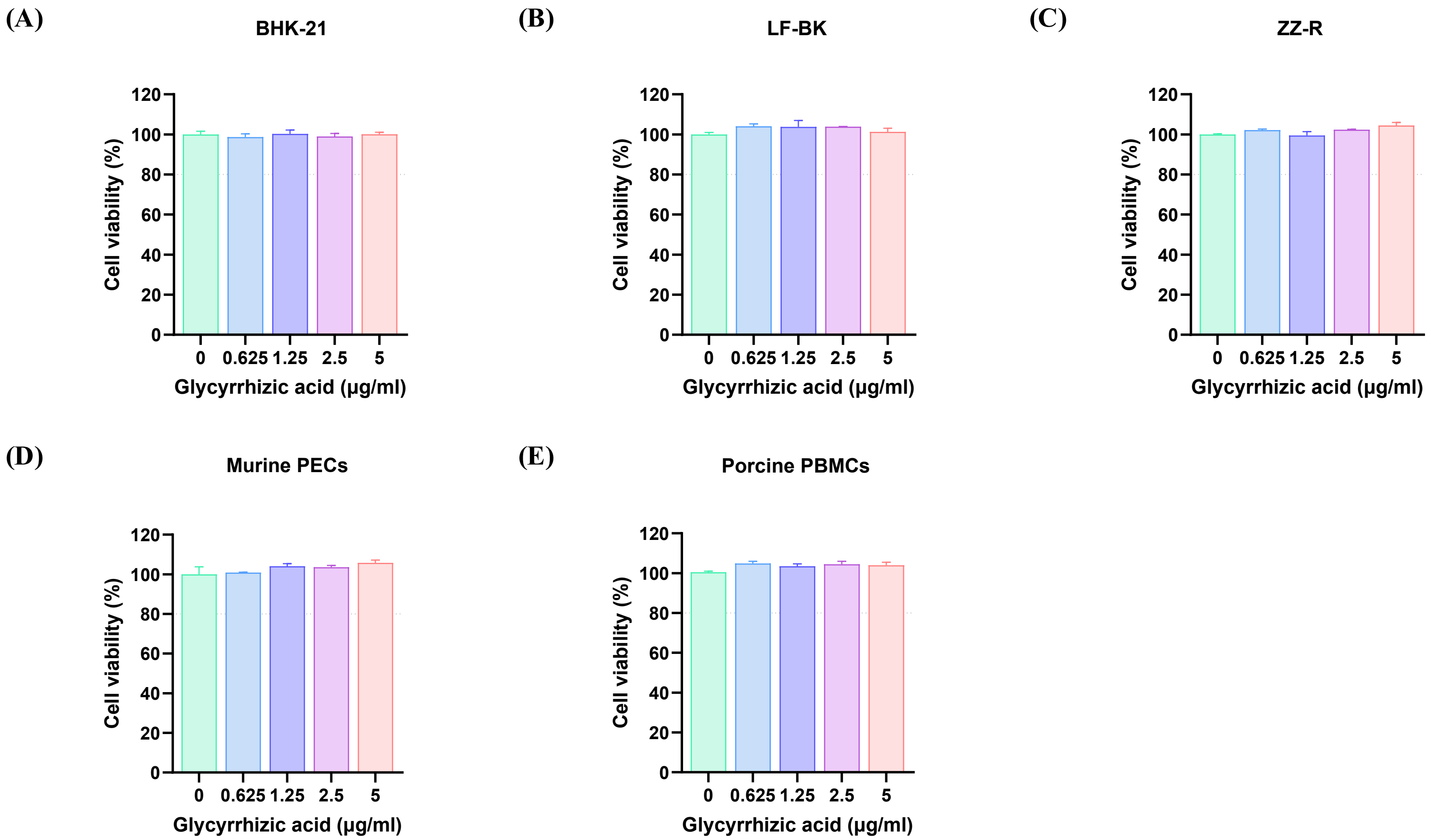

Supplementary Figure 1. Supplementary Figure 1. Cytotoxicity of glycyrrhizic acid measured by cell viability assay in BHK-21, LF-BK, ZZ-R, murine PECs and porcine PBMCs.

(A–E) Cell viability of BHK-21 (A); LF-BK (B), ZZ-R cells (C); murine PECs (D); and porcine PBMCs (E).

Data have been represented as the mean ± SEM of triplicate measurements (*n*=3/group). Statistical analyses were performed using one-way ANOVA with Dunnett's *post hoc* test.

(A)

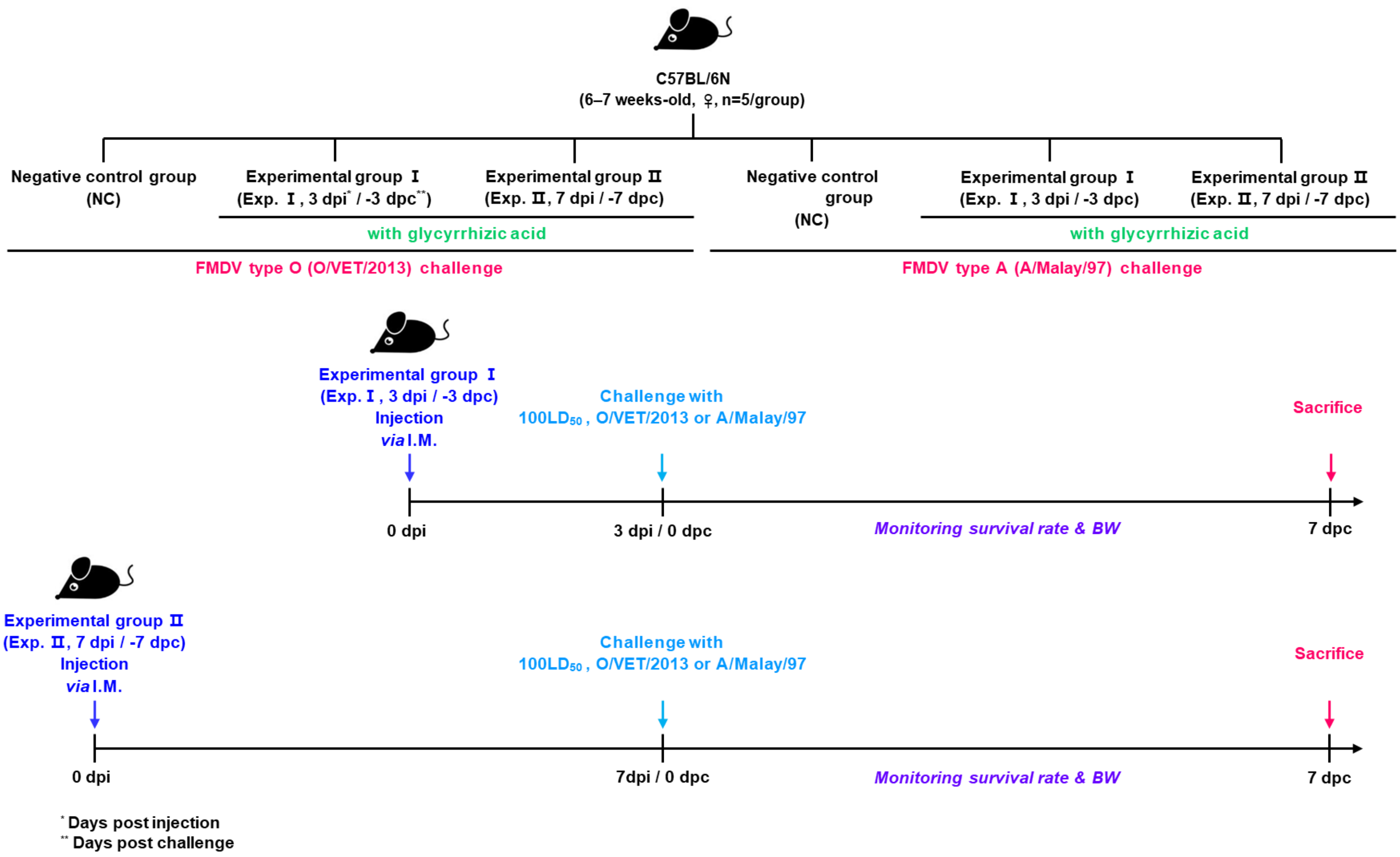
C57BL/6N
(6–7 weeks-old, ♀, n=5/group)
Negative control group
(NC)
Experimental group Ⅰ
(Exp. Ⅰ, 3 dpi* / -3 dpc**)
Experimental group Ⅱ
(Exp. Ⅱ, 7 dpi / -7 dpc)
Negative control group
(NC)
Experimental group Ⅰ
(Exp. Ⅰ, 3 dpi / -3 dpc)
Experimental group Ⅱ
(Exp. Ⅱ, 7 dpi / -7 dpc)
with glycyrrhizic acid
with glycyrrhizic acid
FMDV type O (O/VET/2013) challenge
FMDV type A (A/Malay/97) challenge
Experimental group Ⅰ
(Exp. Ⅰ, 3 dpi / -3 dpc)
Injection
via I.M.
Challenge with
100LD50, O/VET/2013 or A/Malay/97
Sacrifice
0 dpi
3 dpi / 0 dpc
Monitoring survival rate & BW
7 dpc
Experimental group Ⅱ
(Exp. Ⅱ, 7 dpi / -7 dpc)
Injection
via I.M.
Challenge with
100LD50, O/VET/2013 or A/Malay/97
Sacrifice
0 dpi
7dpi / 0 dpc
Monitoring survival rate & BW
7 dpc
* Days post injection
** Days post challenge

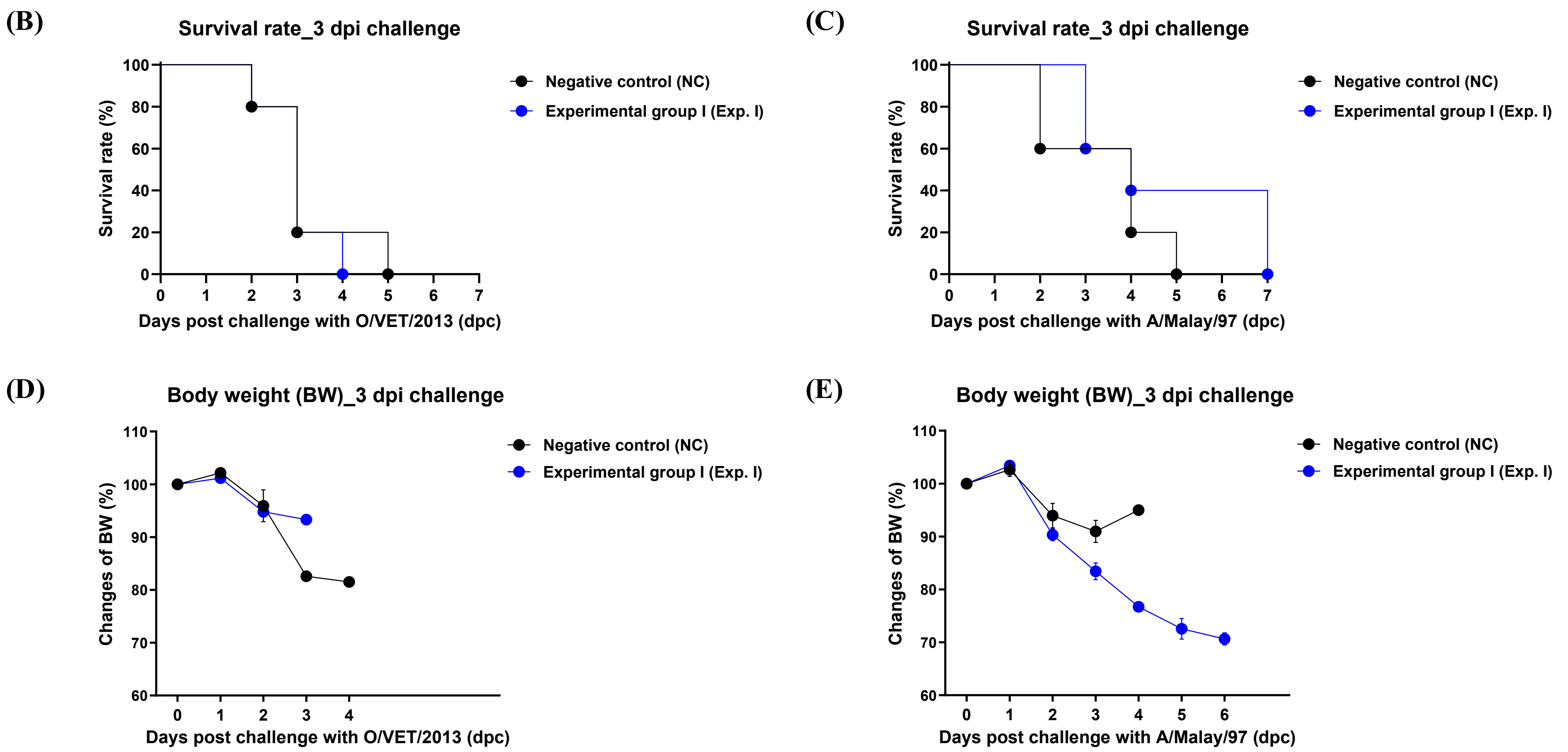
(B)
Survival rate_3 dpi challenge
Survival rate (%)
Days post challenge with O/VET/2013 (dpc)
Negative control (NC)
Experimental group I (Exp. I)
(C)
Survival rate_3 dpi challenge
Survival rate (%)
Days post challenge with A/Malay/97 (dpc)
Negative control (NC)
Experimental group I (Exp. I)
(D)
Body weight (BW)_3 dpi challenge
Changes of BW (%)
Days post challenge with O/VET/2013 (dpc)
Negative control (NC)
Experimental group I (Exp. I)
(E)
Body weight (BW)_3 dpi challenge
Changes of BW (%)
Days post challenge with A/Malay/97 (dpc)
Negative control (NC)
Experimental group I (Exp. I)

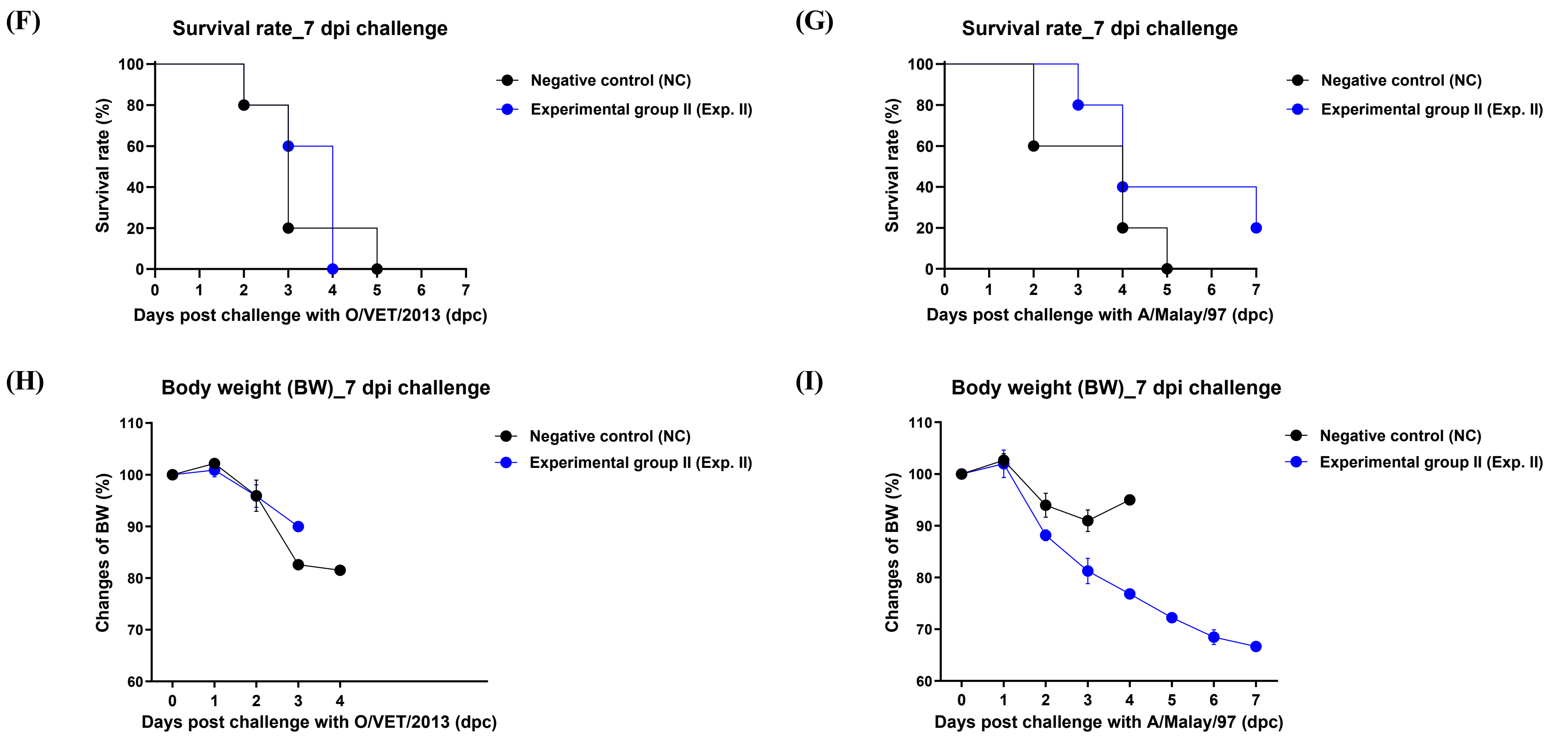

Supplementary Figure 2. Glycyrrhizic acid alone-mediated host defense in early stage of FMDV infection on mice.

C57BL/6 mice (6–7 weeks-old, *n* = 5/group) were administered intramuscularly an glycyrrhizic acid alone. Mice were challenged with FMDV O (100 LD_{50} O/VET/2013) or FMDV A (100 LD_{50} A/Malay/97) at 3 or 7 days post-injection (dpi) using an intraperitoneal injection.

Survival rates and body weights were monitored for 7 days post-challenge (dpc) with the respective viruses. (A–E) experimental workflow (A); survival rates in 3 dpi challenged group with O/VET/2013 (B) or A/Malay/97 (C); changes in body weight 3 dpi challenged group with O/VET/2013 (D) or A/Malay/97 (E); survival rates in 7 dpi challenged group with O/VET/2013 (F) or A/Malay/97 (G); and changes in body weight 7 dpi challenged group with O/VET/2013 (H) or A/Malay/97 (I). Data are presented as mean ± SEM of triplicate measurements (n = 5/group).

1.2 Supplementary Table

Supplementary Table 1. List of primer sequences for qRT-PCR.

Target	Forward/Reverse	Sequence (5'- 3')	Length (mer)
RIG-I	RIG-I F	GCACCTCATACTTACAGCCCA	21
	RIG-I R	CCACAACCAGTAGGAGCACAT	21
SIRT1	SIRT1 F	TGCACGATGGTCTCAGCTTT	20
	SIRT1 R	CAGCAGTGGGACCAAGAAGT	20
MyD88	MyD88 F	CCATTCGAGATGACCCCCTG	20
	MyD88 R	TGCACAAACTGGGTATCGCT	20
TRAF6	TRAF6 F	TCGCAGTAGCTCCTGTACCT	20
	TRAF6 R	AGCTCCCGGATTTGATGGTC	20
NF-κB	NF-κB F	TCGCTGCCAAAGAAGGACAT	20
	NF-κB R	AGCGTTCAGACCTTCACCGT	20
STAT1	STAT1 F	TGCACGATGGTCTCAGCTTT	20
	STAT1 R	CAGCAGTGGGACCAAGAAGT	20
STAT4	STAT4 F	ACATGTCAAAGCCATGTCCA	20
	STAT4 R	ATGTGACAGCCCTCATTTCC	20
CD80	CD80 F	TCAGACACCCAGGTACACCA	20
	CD80 R	GACACATGGCTTCTGCTTGA	20
CD86	CD86 F	TTTGGCAGGACCAGGATAAC	20
	CD86 R	GCCCTTGTCCTTGATTTGAA	20
CD28	CD28 F	TCAAAGGAGTTCCGGGCATC	20
	CD28 R	CTGAAGCAGGCGGGAGTAAT	20
CD19	CD19 F	GGACGACAGACTTCCTGAGC	20
	CD19 R	GTTCTGGCCCATCAGGATTA	20
CD21	CD21 F	TGCCATGCCTACAAAGCTGA	20
	CD21 R	GTAGTAACCAGGGCGGCATT	20
CD81	CD81 F	TCAACAAGGACCAGATCGCC	20
	CD81 R	GAGCGTCTCGTGGAAAGTCT	20

Supplementary Table 1 (continued)

Target	Forward/Reverse	Sequence (5'- 3')	Length (mer)
C3d	C3d F	ACAAATTGACCCAGCGTAGG	20
	C3d R	GCACGTCCTTGCTGTACTGA	20
TNFα	TNFα F	CCCCCAGAAGGAAGAGTTTC	20
	TNFα R	CGGGCTTATCTGAGGTTTGA	20
IFNα	IFNα F	CATCTGCTCTCTGGGCTGTG	20
	IFNα R	TGAGGGGATCCAAAGTCCCT	20
IFNβ	IFNβ F	TGCAACCACCACAATTCCAGA	21
	IFNβ R	GGTTTCATTCCAGCCAGTGC	20
IFNγ	IFNγ F	GCCATTCAAAGGAGCATGGAT	21
	IFNγ R	CTGATGGCTTTGCGCTGGAT	20
IL-1β	IL-1β F	AGCCAGTCTTCATTGTTCAGGT	22
	IL-1β R	TCATCTCTTTGGGGCCATCAG	21
IL-6	IL-6 F	CTGCAGTCACAGAACGAGTG	20
	IL-6 R	CGGCATCAATCTCAGGTGCC	20
IL-12p40	IL-12p40 F	GGAGTATAAGAAGTACAGAGTGG	23
	IL-12p40 R	GATGTCCCTGATGAAGAAGC	20
IL-23p19	IL-23p19 F	CCATATCCAGTGCGGGGATG	20
	IL-23p19 R	AGGCCTTGGTGGATCCTTTG	20
IL-23R	IL-23R F	TCCCTCATTGCAAAGCACAA	20
	IL-23R R	GCATCTCCTCTTGCAAGCAAAT	22
IL-17A	IL-17A F	CTCGTGAAGGCGGGAATCAT	20
	IL-17A R	GGTGTGCTCCGGTTCAAGAT	20
HPRT	HPRT F	CCCAGCGTCGTGATTAGTGA	20
	HPRT R	GCCGTTCAGTCCTGTCCATA	20
